# Unraveling Tissue‐Specific Fatty Acid Biosynthesis and Inter‐Tissue Crosstalk in Mice through Stable‐Isotope Tracing Metabolomics

**DOI:** 10.1002/advs.202503662

**Published:** 2025-05-30

**Authors:** Beizi Xing, Ruohong Wang, Tianzhang Kou, Wenbin Liu, Zheng‐Jiang Zhu

**Affiliations:** ^1^ Interdisciplinary Research Center on Biology and Chemistry Shanghai Institute of Organic Chemistry Chinese Academy of Sciences Shanghai 200032 China; ^2^ University of Chinese Academy of Sciences Beijing 100049 China; ^3^ Shanghai Key Laboratory of Aging Studies Shanghai 201210 China

**Keywords:** fatty acid biosynthesis, inter‐tissue crosstalk, mass spectrometry, metabolomics, stable‐isotope tracing

## Abstract

Biosynthesis of free fatty acids (FFAs) in mammals is pivotal for metabolic homeostasis, yet a comprehensive understanding of tissue‐specific biosynthesis and inter‐tissue crosstalk of FFAs remains incomplete. In vivo stable‐isotope tracing metabolomics is utilized to comprehensively measure FFA biosynthesis and inter‐tissue crosstalk in mice. Systematically assessing tissue‐specific biosynthesis of 13 FFAs across 15 tissues unveils dynamic spatial and temporal accumulation and redistribution of FFAs throughout the body. Employing an analytical framework to deconvolve mass isotopologue patterns, inter‐tissue crosstalk is explored for saturated, polyunsaturated, and monounsaturated FFAs, and quantify communications of FFA (16:0) and FFA (18:0) between the liver and other tissues. Then, a decline in fatty acid biosynthesis in peripheral tissues but not in the brain of aged mice is observed, particularly evident in palmitic acid and monounsaturated fatty acids. These findings illuminate the complex interplay between tissue‐specific fatty acid biosynthesis and the maintenance of metabolic homeostasis.

## Graphical abstract

Biosynthesis rates of 13 FFAs are quantitatively calculated in 15 tissues from glucose and an analytical framework to quantitatively calculate inter‐tissue communications of fatty acids between the liver and other tissues is developed. Moreover, the approach is extended to aging condition and a system‐wide decline of fatty acid synthesis in peripheral tissues but not in the brain of aged mice is revealed.

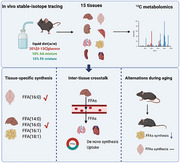

## 1 Introduction

Fatty acids play crucial roles in maintaining metabolic homeostasis, serving as major sources of energy, components of membrane lipids and important signaling molecules.^[^ ^1^ ^]^ Mammals possess the ability to synthesize a diverse array of fatty acids, varying in chain lengths and degrees of unsaturation. De novo fatty acid synthesis in mammals involves the consumption of acetyl‐CoA to produce palmitic acid (PA), along with small amounts of myristic acid and stearic acid, catalyzed by fatty acid synthase (FASN) and acetyl‐CoA carboxylase 1 (ACC1). Subsequently, long chain fatty acid elongases (ELOVL), fatty acid desaturases (FADS) and stearoyl‐CoA desaturase 1 (SCD1) further catalyze these products, increasing their carbon chain length and degree of unsaturation, resulting in a variety of fatty acids. The fatty acid biosynthesis in mammals, integral to de novo lipogenesis (DNL), is essential for the organismal functions. Fatty acid biosynthesis is an energetically demanding process and must be tightly regulated to ensure the balance between the availability of fatty acids and the functional and energetic requirements of diverse cells and tissues.^[^ ^2^ ^]^ Liver and adipose are the main tissues for biosynthesis of fatty acids. The deregulation of fatty acid metabolism in liver and adipose tissue can disrupt lipid homeostasis in the body, leading to various human diseases such as non‐alcoholic fatty liver disease (NAFLD), obesity and insulin resistance.^[^ ^3^ ^]^ However, evidence indicates that many other tissues possess the capability to synthesize fatty acids,^[^ ^4^ ^]^ which maintains metabolic homeostasis. For instance, fatty acid synthesis in muscle contributes to the equilibrium between insulin sensitivity and muscle strength by leveraging calcium signaling.^[^ ^5^ ^]^ Fatty acid synthesis in the brain is involved in regulating food intake and is reported to be active in the hypothalamus, a specialized region within the central nervous system (CNS) responsible for orchestrating the regulation of energy homeostasis.^[^ ^4b^ ^]^ Consequently, quantifying the tissue‐specific activity for fatty acid biosynthesis is essential to understand the physiological significance of fatty acid metabolism across various tissues.

During the postprandial state, mammals convert excess carbohydrates from the diet into stored energy through fatty acid biosynthesis. This process is pivotal in both glucose and lipid metabolism, especially in adapting to sudden increases after carbohydrate intake.^[^ ^3^, ^6^ ^]^ Liver and adipose tissue are the primary sites for this biosynthetic pathway. Subsequently, inter‐tissue crosstalk takes place, encompassing uptake, synthesis, and secretion of fatty acids across various tissues, to maintain metabolic homeostasis. Dysregulation of inter‐tissue crosstalk can lead to adverse effects such as the accumulation of ectopic lipids and dysfunction of specific tissues.^[^ ^2^, ^6^, ^7^ ^]^ For instance, oleic acid functions as a signaling molecule regulating fatty acid uptake and oxidation in multiple tissues.^[^ ^2b^ ^]^ Cardiomyocyte‐specific overexpression of FATP (fatty acid transporter protein), a protein involved in fatty acid uptake, has been shown to cause the accumulation of fatty acids in the heart, leading to cardiovascular disease in mice.^[^ ^8^ ^]^ Additionally, lipid accumulation in the kidney and bones may contribute to disturbances in whole‐body glucose metabolism.^[^ ^9^ ^]^ Therefore, it is crucial to further investigate the systemic regulation of newly synthesized fatty acid crosstalk across multiple tissues at the whole‐body level. However, the redistribution of newly synthesized fatty acids through inter‐tissue crosstalk after dietary intake to meet the functional and energetic needs of tissues remains unclear.

In this work, we designed an in vivo stable‐isotope tracing metabolomics approach by administering [U‐^13^C]‐glucose in diet to comprehensively track multi‐tissue fatty acid biosynthesis and inter‐tissue crosstalk in mice. We first elucidated distinct accumulation of newly synthesized free fatty acids (FFAs) in different tissues, revealing a spatiotemporal shift and crosstalk of fatty acid synthesis across 15 different tissues during the postprandial state. We further quantified tissue‐specific heterogeneity of fatty acid synthesis rates in mice. Then, we demonstrated different inter‐tissue metabolic crosstalk patterns for saturated, polyunsaturated and monounsaturated FFAs, and further quantified the crosstalk of specific FFAs (16:0) and (18:0) between liver and other tissues. Additionally, our investigation extended to aged mice, where we observed a decline in fatty acid biosynthesis in peripheral tissues, with minimal changes detected in brain tissue. In summary, our system‐wide quantitative analysis of fatty acid biosynthesis and inter‐tissue crosstalk in mice sheds light on the intricate interplay between dietary glucose utilization, tissue‐specific fatty acid biosynthesis, and the maintenance of metabolic homeostasis.

## 2 Results

### 2.1 Contribution of Dietary Glucose to Fatty Acids Biosynthesis in Mouse Tissues

**Figure 1:**
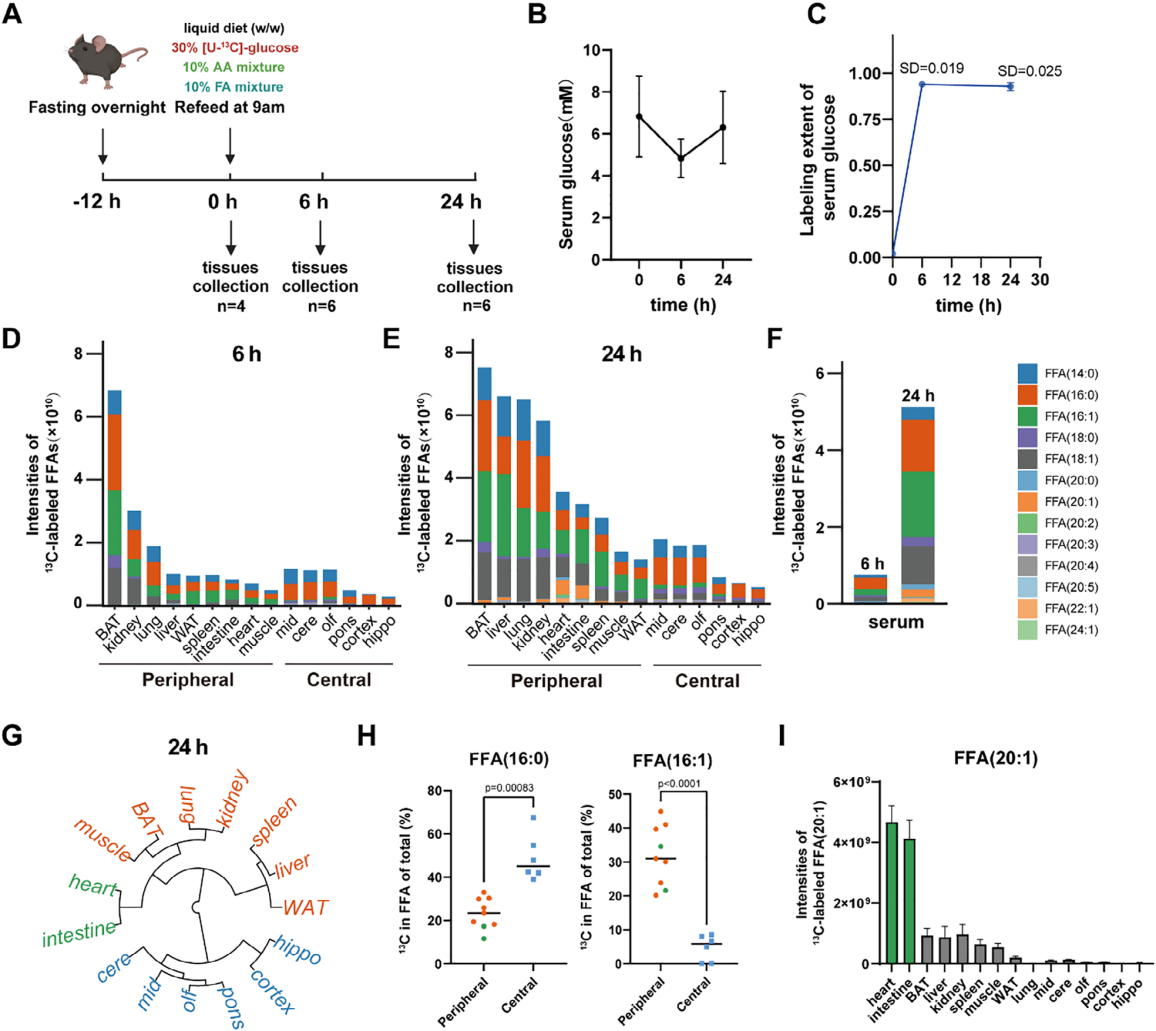
Contribution of dietary glucose to fatty acids biosynthesis in mouse tissues. A) Schematic illustration of in vivo stable‐isotope tracing metabolomics experiment in mice (n = 4 at 0 h, n = 6 at 6 and 24 h; 12‐week‐old; C57BL/6J, male). B) Glucose concentrations (mm) in mouse serum measured at 0, 6, and 24 h after the liquid diet refeeding. Error bars represent the standard deviation (SD) of mean. C) Labeling extents of glucose in serum measured by LC‐MS at 0, 6, and 24 h after labeling. The labeling extent was calculated by Equation (2) in Experimental Section. Error bars represent the standard deviation (SD) of mean. D–F) Intensities of ^13^C‐labeled FFAs in 15 tissues and serum at 6 and 24 h after labeling. Intensities of ^13^C‐labeled FFAs were calculated by Equation (1) in Experimental Section. BAT, brown adipose tissue; WAT, white adipose tissue; intestine, small intestine; mid, midbrain; cere, cerebellum; olf, olfactory bulb; hippo, hippocampus. G) Hierarchical clustering analysis of peripheral and central tissues was performed using the fractions of each ^13^C‐labeled FFA relative to the total intensities of labeled FFAs in each tissue after 24 h labeling. H) The percentages of ^13^C‐labeled FFA (16:0) and FFA (16:1) relative to their total intensities in peripheral and central tissues after 24 h refeeding. The p‐value is calculated by two‐sided Student's t‐test. I) Intensities of ^13^C‐labeled FFA (20:1) in different tissues after 24 h labeling. Error bars represent the standard deviation (SD) of mean.

Fatty acid biosynthesis plays a pivotal role in the metabolic adaption from fasting to feeding, maintaining metabolic homeostasis and organismal functions. To investigate the biosynthesis and accumulation of newly synthesized FFAs in mice, we designed an in vivo stable‐isotope tracing metabolomics experiment. Mice underwent a 12‐h fasting period followed by re‐feeding with a liquid diet containing 30% [U‐^13^C]‐glucose, 10% protein, and 10% fatty acid mixture for 6 and 24 h, respectively (**Figure** **1**A). Upon entering the mouse body, ^13^C‐labeled glucose undergoes glycolysis, yielding acetyl‐CoA, a key precursor for fatty acid synthesis. By monitoring the incorporation of the ^13^C label into fatty acids, we could trace the dynamic process of fatty acid synthesis within the organism. Subsequently, we harvested 15 tissues and serum at each time point for LC‐MS‐based untargeted metabolomic profiling. Throughout the experiment, the glucose concentrations in mouse serum remained within physiologically normal ranges (Figure 1B). Additionally, we calculated the labeling extent of glucose in serum (Equation (2)) which remained stable after 6 h, indicating the achievement of isotopic steady state for glucose (Figure 1C). Moreover, the intra‐group variability in serum glucose labeling was ≈2%, supporting that inter‐individual differences in absorption had a negligible impact on overall labeling efficiency. Next, we measured the intensities of ^13^C‐labeled fatty acids in all collected tissue samples using LC‐MS. A total of 21 free fatty acids were measured (Figure S1, Supporting Information). Among these, 13 fatty acids were labeled in over 40% of tissues, encompassing saturated fatty acids (SFAs), monounsaturated fatty acids (MUFAs), and polyunsaturated fatty acids (PUFAs) with different intensities and ^13^C enrichments (Figure S2A,B, Supporting Information). For synthetically active fatty acids, such as FFA (16:0), ≈20–40% are newly synthesized, while for synthetically inactive fatty acids, such as PUFAs, less than 10% are derived from biosynthesis (Figure S3, Supporting Information). We further calculated the intensities of ^13^C‐labeled FFAs in different tissues and serum, which reflected the contribution of [U‐^13^C]‐glucose from the diet to newly synthesized FFAs (Figure 1D,E and Equation (1)). Additionally, our data revealed differential labeling extents of glucose among various tissues, reflecting distinct labeled glucose uptake and usage (Figure S2C,D, Supporting Information). It is important to note that the tissue‐specific utilization of glucose should be considered a factor contributing to the heterogeneity in fatty acid biosynthesis. In this context, brown adipose tissue (BAT) exhibited the highest level of ^13^C‐labeled FFAs after 6 h of refeeding, indicating its heightened FFA synthesis activity using dietary glucose (Figure 1D). Furthermore, after 24 h of refeeding, the liver, lung, and kidney demonstrated comparable levels of ^13^C‐labeled FFAs compared to BAT (Figure 1E). Levels of ^13^C‐labeled FFAs in serum were 5–6 times higher at 24 h than at 6 h of refeeding, indicating synchronous accumulation of the newly synthesized FFAs in both serum and tissues (Figure 1F). These findings revealed a spatiotemporal accumulation of newly synthesized FFAs across different tissues during the postprandial state. Additionally, most peripheral organs exhibited higher levels of ^13^C‐labeled FFAs compared to central organs, such as various brain regions. This observation is consistent with the brain's predominant reliance on glucose for energy metabolism rather than active participation in fatty acid synthesis.^[^ ^10^ ^]^ Among the 13 FFAs analyzed, FFA (14:0), FFA (16:0), FFA (16:1), and FFA (18:1) emerged as the predominant newly synthesized FFA species present in tissues and in circulatory serum (Figure 1D–F).

We further investigated the accumulation patterns of newly synthesized FFAs across multiple tissues. The fractions of ^13^C‐labeled FFAs to the total intensities of labeled FFAs in each tissue were used for hierarchical clustering analysis (HCA), resulting in two cluster groups: cluster 1 comprising all nine peripheral tissues and cluster 2 including six brain regions (Figure 1G). Particularly noteworthy was the preference of brain regions for synthesizing FFA (16:0), accounting for ≈50% of the newly synthesized FFAs (Figure 1H). In contrast, peripheral tissues showed a higher propensity for synthesizing FFA (16:1), representing ≈30% of newly synthesized FFAs (Figure 1H). Furthermore, we observed tissue‐specific accumulation of newly synthesized FFA (20:1) in the heart and small intestine, significantly less pronounced in other tissues, potentially reflecting the unique functions of these tissues (Figure 1I). In conclusion, these findings highlight the unique accumulation patterns of newly synthesized FFAs in various tissues, indicating a dynamic spatial and temporal redistribution, as well as inter‐tissue communication, of fatty acid synthesis in mice during the postprandial state.

### 2.2 Tissue‐Specific Heterogeneity of Fatty Acid Synthesis and Accumulation in Mice

**Figure 2:**
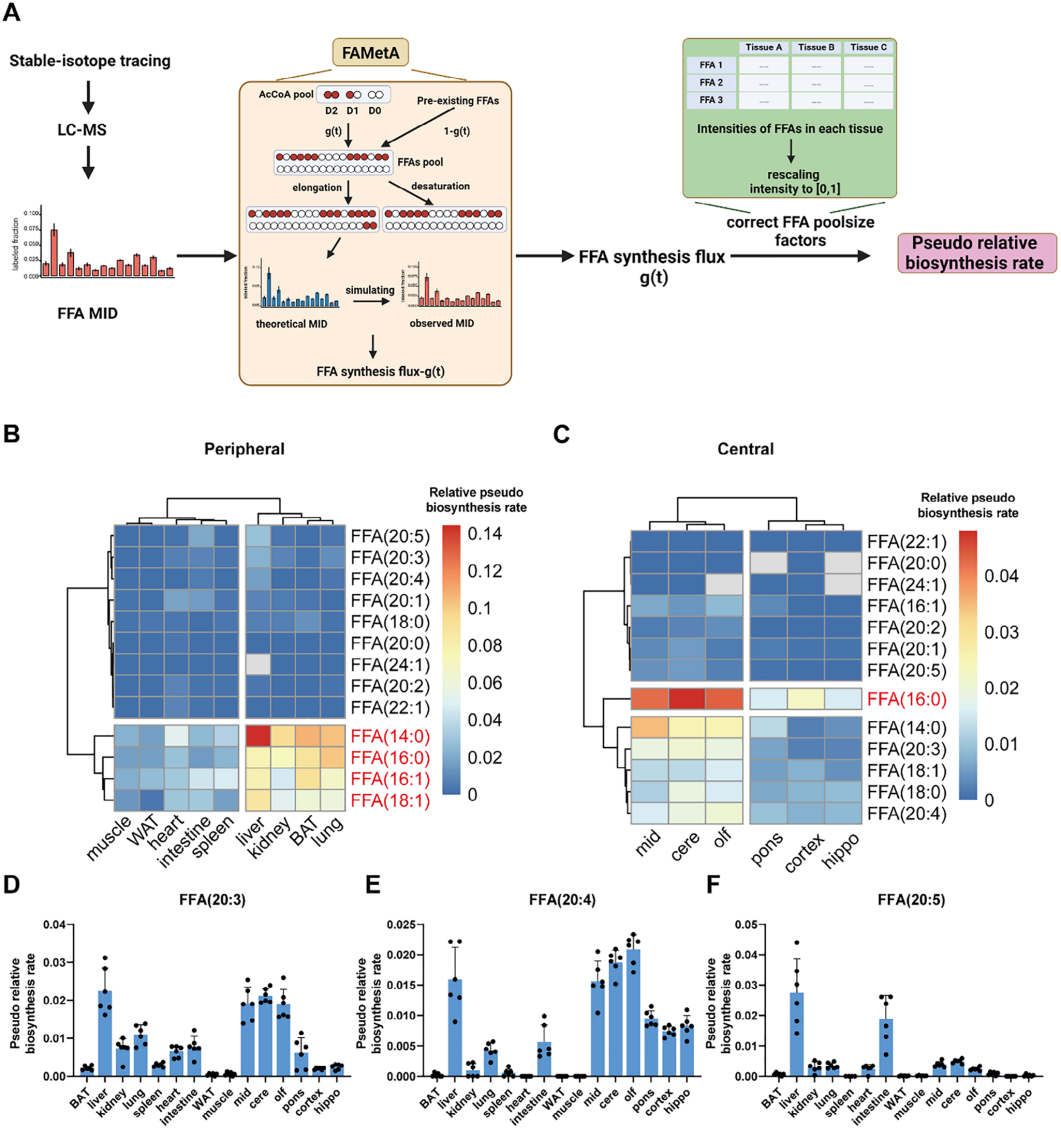
Tissue‐specific heterogeneity of fatty acid synthesis and accumulation in mice. A) The workflow to calculate the pseudo relative biosynthesis rates across tissues in mice using FAMetA. The MID of each FFA measured by LC‐MS was inputted into FAMetA. Here, g(t) represents the synthesis flux, or the fraction of newly synthesized FFAs during labeling time t. D0, D1, D2 represent the labeled fractions of M+0, M+1 and M+2 isotopologues of acetyl‐CoA. The outputted synthesis flux values were further corrected by the FFA pool size to obtain the pseudo relative synthesis rates. B,C) Heatmaps show pseudo relative biosynthesis rates of FFAs in peripheral and central tissues, respectively, after 24 h refeeding. The colors show the average values of pseudo relative biosynthesis rates in biological samples (n = 6 mice). Gray indicates the failed calculation due to the low fractions of ^13^C‐labeled FFAs. D–F) Bar plots show the pseudo relative biosynthesis rates of FFA (20:3), FFA (20:4), FFA (20:5) in 15 tissues after 24 h refeeding related to (B) and (C). Error bars represent the standard deviation (SD) of mean (n = 6 mice; 12‐week‐old; C57BL/6J; male).

We observed different accumulation patterns and rates of newly synthesized FFAs across tissues between 6 and 24 h of refeeding (Figures S2A,B and S3, Supporting Information). The accumulation of ^13^C FFAs in brown and white adipose tissues almost did not increase from 6 to 24 h of refeeding which indicates the rapid regulatory role of adipose tissues in glucose metabolism through fatty acid biosynthesis. In contrast, the process in other tissues appears to be more sustained until 24 h of refeeding, responding to the demand for fatty acids. Additionally, the labeling extents of different fatty acids vary among tissues, indicating the heterogeneity in the accumulation rates of newly synthesized fatty acid synthesis across tissues. To further evaluate tissue‐specific heterogeneity of synthesis and accumulation of newly synthesized FFAs in mice, we utilized FAMetA^[^ ^11^ ^]^ to calculate the pseudo biosynthesis rates of 13 FFAs across 15 tissues using experimental mass isotopologue distribution (MID) measured by LC‐MS. In this analysis, we assumed that the measured ^13^C‐labeled FFAs were all synthesized within the respective tissues. It is important to note that the labeled FFA pools in tissue could result from both uptake and in situ synthesis. Consequently, the calculated “pseudo biosynthesis rates” are likely to be higher than the actual biosynthesis rates due to the contribution from uptake. In practice, FAMetA employs a mathematical algorithm to fit theoretical MID to experimental MID of FFAs, iteratively adjusting model parameters such as labeling fractions of precursor acetyl‐CoA isotopologues (D2, D1, D0) and FFA synthesis flux (g(t)). Subsequently, we corrected FFA pool size factors to generate pseudo relative biosynthesis rates of FFAs in different tissues (**Figure** **2**A).

In peripheral tissues, FFA (14:0), FFA (16:0), FFA (16:1), and FFA (18:1) displayed significantly higher biosynthesis rates (Figure 2B; Figure S4A–I, Supporting Information), affirming their status as major FFA species synthesized. Notably, the liver, kidney, BAT, and lung exhibited higher biosynthesis rates of FFAs than other peripheral tissues (Figure 2B). Across different brain tissues, FFA (16:0) emerged as the major FFA synthesized in the brain (Figure 2C; Figure S4A–I, Supporting Information), consistent with the previous findings in Figure 1H. Interestingly, despite the physiological proximity of central regions, heterogeneity in fatty acid synthesis and accumulation was observed among different brain zones. Specifically, the midbrain, cerebellum, and olfactory bulb demonstrated faster biosynthesis rates of fatty acids compared to the hippocampus, cortex, and pons (Figure 2C). To evaluate whether this heterogeneity could be attributed to differences in glucose uptake and utilization, we assessed the expression of glucose transporter genes (*GLUT1* and *GLUT3*) and quantified the labeling extent of pyruvate across brain regions. Although variations in glucose transporter expression were detected (Figure S4J,K, Supporting Information), the downstream labeling of pyruvate was largely consistent across regions, with the exception of the cortex (Figure S4L, Supporting Information), which indicated glucose transport activity did not substantially influence downstream ^13^C labeling of metabolites. These results support the notion that the heterogeneity in fatty acid synthesis reflects intrinsic regional metabolic differences within the brain. Regarding the synthesis of PUFAs in peripheral tissues, such as FFA (20:3), FFA (20:4), and FFA (20:5), the liver exhibited the highest activity in PUFA synthesis (Figure 2D–F), while other peripheral tissues displayed comparatively lower levels of PUFA synthesis. In the brain, FFA (20:3) and FFA (20:4) were prominently synthesized (Figure 2D,E). This was consistent with the facts that PUFAs and their mediators regulate several processes within the brain, such as neurotransmission, cell survival and neuroinflammation, and thereby mood and cognition.^[^ ^12^ ^]^ Together, these results confirmed the tissue‐specific heterogeneity of fatty acid synthesis and accumulation in mice.

### 2.3 Metabolic Crosstalk of FFAs between the Circulatory Serum and Tissues

**Figure 3:**
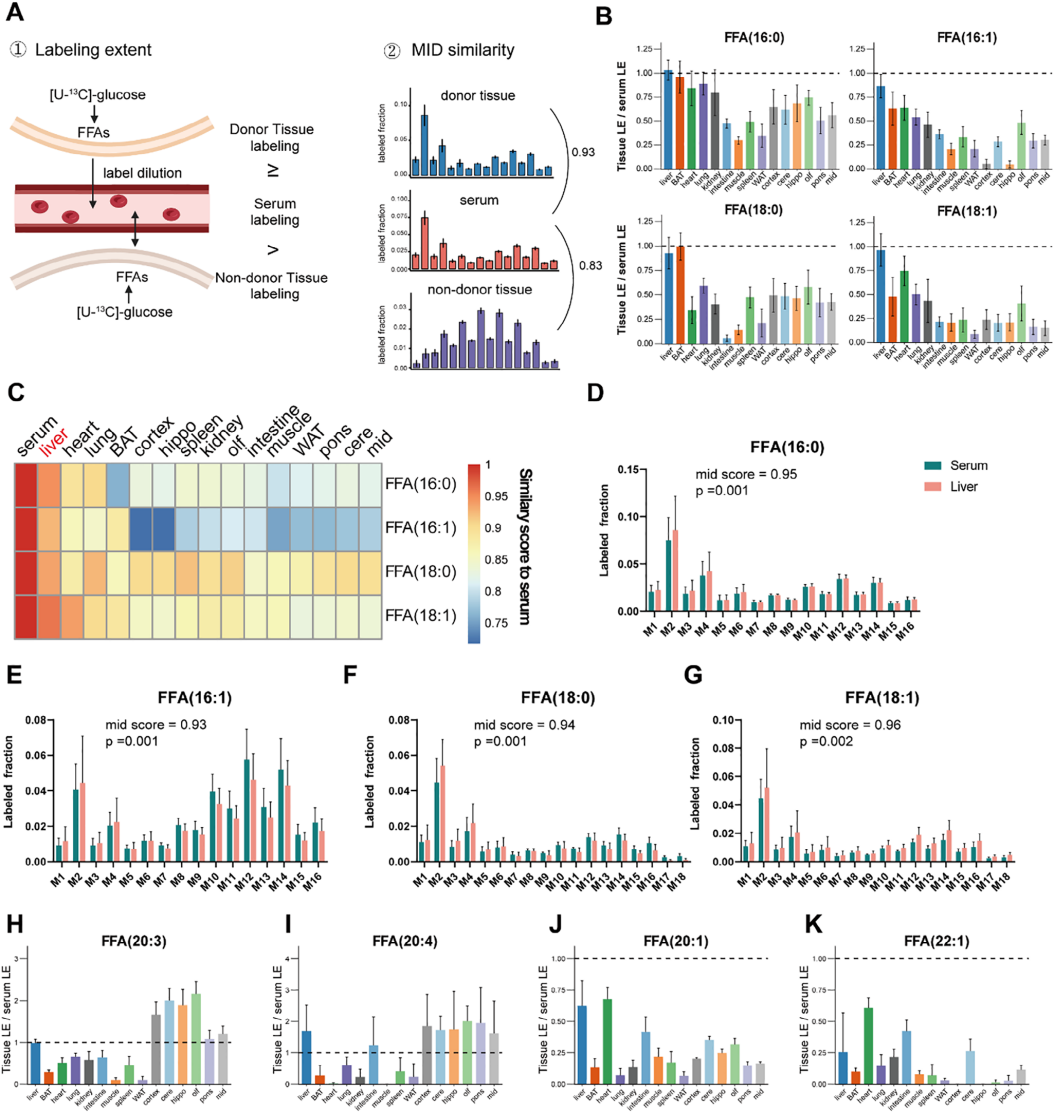
Metabolic crosstalk of FFAs between the circulatory serum and tissues. A) Two principles to determine donor tissues of FFAs in circulatory serum. The MID similarity scores were calculated by manhattan distance (Equation (4)). MID, mass isotopologue distribution. B) Comparisons of labeling extents of FFA (16:0), FFA (16:1), FFA (18:0), FFA (18:1) in different tissues to those in serum after 24 h labeling. Error bars represent the standard deviation (SD) of mean (n = 6 mice; 12‐week‐old; C57BL/6J; male). C) A heatmap shows MID similarity scores of FFA (16:0), FFA (16:1), FFA (18:0), FFA (18:1) between each tissue and serum after 24 h labeling. The colors represent the median values of MID similarity scores in biological samples (n = 6 mice). The serum samples serve as a reference. D–G) MID of FFA (16:0), FFA (16:1), FFA (18:0), FFA (18:1) in serum and liver after 24 h labeling. Error bars represent the standard deviation (SD) of mean (n = 6 mice). The p‐value was calculated by Monte Carlo permutation test. H‐K) Comparisons of labeling extents of FFA (20:3), FFA (20:4), FFA (20:1) and FFA (22:1) in different tissues to those in serum after 24 h labeling. Error bars represent the standard deviation (SD) of mean (n = 6 mice; 12‐week‐old; C57BL/6J; male).

The inter‐tissue metabolic crosstalk of fatty acids plays a crucial role in maintaining overall metabolic homeostasis and energy balance in the body. Fatty acids synthesized in one tissue are transported to and utilized by other tissues for various purposes such as energy production, storage, or structural functions. Given the central role of the circulatory system in facilitating this metabolic communication, we sought to characterize the sources of FFAs in circulatory serum. To achieve this, we established two fundamental principles: 1) FFAs originating from the donor tissue should exhibit higher labeling extents compared to those in the circulatory serum due to the dilution of ^13^C labeling. Once the exchange between the donor tissue and serum becomes stable, the labeling extents of FFAs in both the serum and donor tissue converge and become similar. 2) The MID of individual FFAs in the donor tissue should closely resemble those in the serum (**Figure** **3**A). Initially, we focused on four high flux‐carrying FFAs, namely FFA (16:0), FFA (18:0), FFA (16:1), and FFA (18:1). We calculated the labeling extents of individual FFAs within tissues relative to those in the serum. Results showed that FFAs in the liver exhibited the higher or similar labeling extents comparable to those observed in the serum (Figure 3B), which indicated that the metabolic exchange between the liver and serum has reached equilibrium. Additionally, we assessed the similarity of MID of individual FFAs between the serum and each tissue. It should be noticed that odd‐numbered mass isotopologues of ^13^C‐labeled FFAs may due to the cleavage of citrate in the cytoplasm which produces M+1 acetyl‐CoA. Our analysis indicated that FFAs from the liver exhibited the most similarities to those from the serum among all tissues (Figure 3C). The specific MIDs of individual FFAs were illustrated in Figure 3D–G. Notably, labeling extents of FFA (16:0) in BAT, heart, lung and kidney and labeling extent of FFA (18:0) in BAT were also similar to those in liver (Figure 3B). However, due to their low MID similarity to serum, we assume that their crosstalk with serum is less than the liver (Figure 3C; Figure S5, Supporting Information). Collectively, our findings suggest that the predominant source of FFAs (16:0), FFA (18:0), FFA (16:1), and FFA (18:1) present in the serum is the liver.

We then expanded our analysis to encompass additional FFAs present in the circulatory serum. Specifically, we observed two distinct categories of FFAs, each exhibiting notably different metabolic crosstalk between tissues and the circulatory system. PUFAs, such as FFA (20:2), FFA (20:3), FFA (20:4), and FFA (20:5) displayed possible contributions from multiple tissues to their pool in the circulatory serum (Figure 3H,I; Figure S6A–C, Supporting Information). Notably, the brain had higher labeling extents of FFA (20:3) and FFA (20:4), suggesting the brain tends to synthesize these two FFAs in situ rather than uptake them from serum (Figure 3H,I). Accordingly, we found that the brain had higher pseudo synthesis rates for FFA (20:3) and FFA (20:4) compared to other tissues (Figure 2D,E). These results indicated the biosynthesis of FFA (20:3) and FFA (20:4) in brain is very active. In contrast, MUFAs, including FFA (20:1), FFA (22:1), and FFA (24:1), demonstrated a relatively distant metabolic connection between tissues we measured and the circulatory serum, as evidenced by their lower labeling extents in tissues compared to those in the serum and insufficient MID similarity (Figure 3J,K; Figure S6D,E, Supporting Information). It is intriguing to observe higher labeling extends of certain MUFAs in serum compared to the tissues. Notably, this discrepancy seems to be more pronounced for very long‐chain MUFAs (C≥20). The results suggested that other tissues (such as skin) might be the source of these circulating MUFAs, although they were not measured in our study. Additionally, FFA (14:0) emerged as a major FFA species synthesized in various organs such as the liver, BAT, lung, kidney and brain, before being released into the circulatory system (Figure S6F–H, Supporting Information). In contrast, FFA (20:0) is identified as a FFA species exhibiting disconnection between tissues we measured and the serum, like MUFAs, with its lower labeling extents and insufficient MID similarity (Figure S6G,H, Supporting Information). In conclusion, our findings identify the liver as the primary source of key FFAs in serum, underscoring its pivotal role in fatty acid metabolism. Additionally, our analysis revealed differing inter‐tissue metabolic crosstalk patterns for PUFAs and MUFAs.

### 2.4 Quantitative Characterization of FFA Metabolic Crosstalk between the Liver and Other Tissues

**Figure 4:**
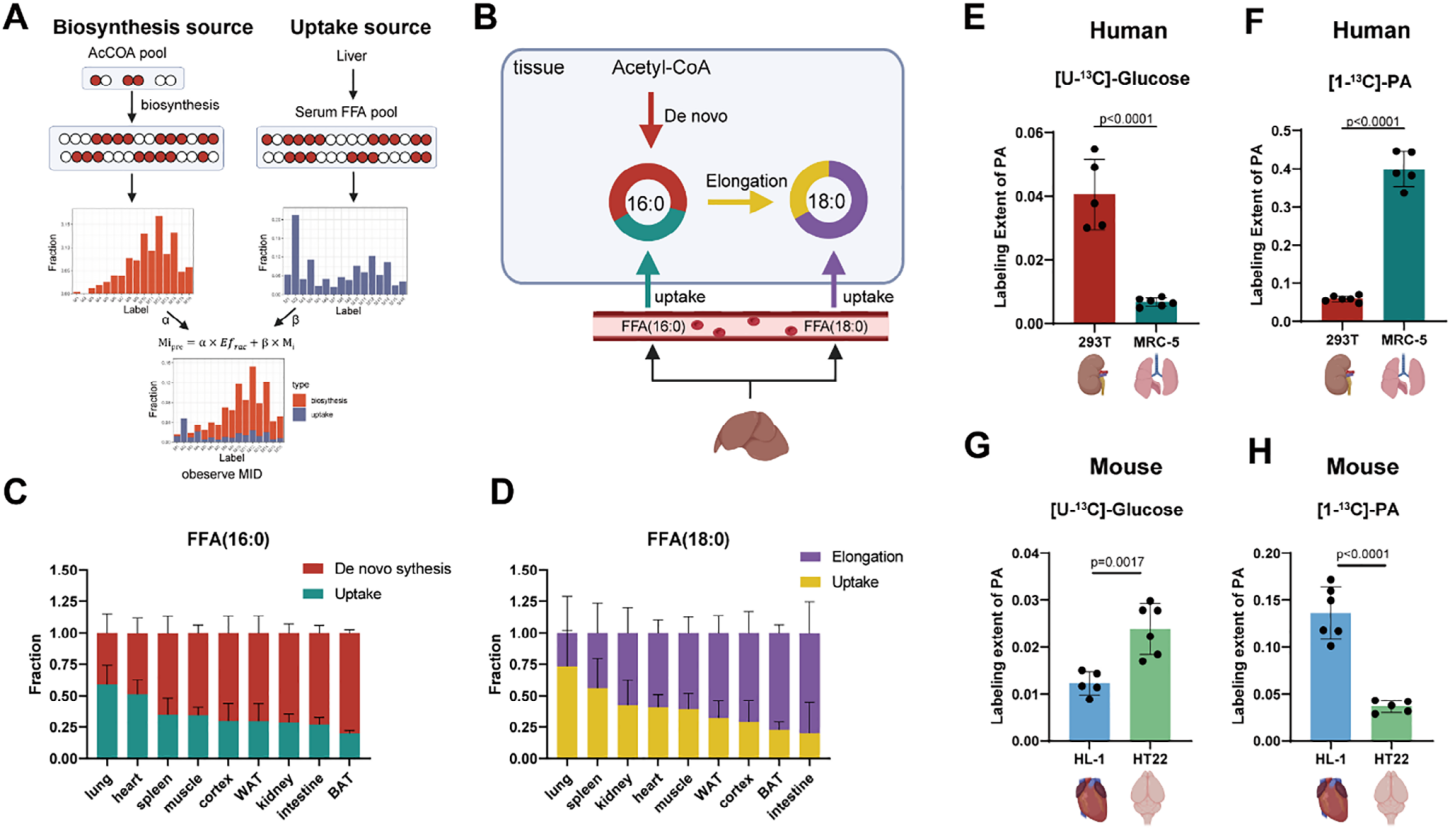
Quantitative characterization of FFA metabolic crosstalk between the liver and other tissues. A) A quantitative model to deconvolve the fraction of synthesis and the uptake fraction of newly synthesized FFAs in tissues. α and β present the fraction of synthesis and the uptake fraction of newly synthesized FFA in tissues, respectively. For FFA (16:0), the model of deconvolution of de novo synthesis and uptake is characterized by Equations (5)–(7). For FFA (18:0), the model of deconvolution of synthesis and uptake is characterized by Equations (8) and (9). B) The proposed synthesis and uptake routes of FFA (16:0) and FFA (18:0) in tissues. Labeled FFA (16:0) in tissues can be taken up from serum or synthesized from the labeled Acetyl‐CoA. Labeled FFA (18:0) in tissues can be taken up from serum or synthesized through the elongation reaction of FFA (16:0). C,D) The calculated compositions of newly synthesized FFA (16:0) and FFA (18:0) in mouse tissues by the quantitative model shown in (a) after 24 h labeling. Error bars represent the standard deviation (SD) of mean (n = 6 mice; 12‐week‐old; C57BL/6J; male). E–H) Labeling extents of FFA (16:0) in 293T, MRC‐5, HL‐1, and HT22 cell lines using [1‐^13^C]‐palmitic acid and [U‐^13^C]‐glucose labeling for 48 h. PA, palmitic acid. 293T, human embryonic kidney 293 cells; MRC‐5, human lung epithelial cells; HL‐1, mouse‐derived cardiac muscle cell line; HT22, immortalized hippocampal cell line. Error bars represent the standard deviation (SD) of mean (n = 5‐6 biologically independent replicates). The p‐value was calculated by two‐sided Student's t‐test.

Both biosynthesis and uptake of FFAs from the circulatory system contribute to FFA levels in tissues. Notably, high flux‐carrying FFAs, such as FFA (16:0) and FFA (18:0), are primarily synthesized in the liver and subsequently released into the serum. Our aim is to further elucidate the metabolic crosstalk of these FFAs between the liver and other peripheral tissues. Interestingly, the same FFA originating from biosynthesis and uptake routes generates different isotopologue patterns (**Figure** **4**A). The integration of isotopologue patterns from two routes, based on their respective contribution fractions, corresponds with the observed isotopologue pattern in the specific tissue. Conversely, by utilizing isotopologue patterns from both biosynthesis and uptake routes, along with the observed isotopologue pattern in the specific tissue, one can mathematically deconvolve the respective contribution fractions of each route. Here, we successfully deconvolved the contribution fractions of biosynthesis and uptake routes for FFA (16:0) and FFA (18:0) in all tissues (Figure 4B–D; Figures S7–S9, Supporting Information). Specifically, uptake fractions were utilized to characterize the relative crosstalk of fatty acid metabolism between each tissue and the liver. Our findings revealed that among the newly synthesized FFA (16:0), 60% of those in the lung were derived from the serum, with a small portion originating from synthesis (Figure 4C). This suggests that the lung predominantly absorbs fatty acids biosynthesized in the liver, establishing inter‐tissue crosstalk with liver fatty acid metabolism. In contrast, tissues such as BAT and the small intestine exhibited lower absorptions of FFA (16:0) from the serum. BAT and the small intestine had approximately 20% of FFA (16:0) derived from the serum, with a larger proportion derived from biosynthesis (Figure 4C). Similar results were obtained for FFA (18:0) (Figure 4D).

To validate the results, we conducted in vitro experiments using cell lines derived from various tissues to assess differences in the preference for fatty acid synthesis and uptake among these cells. Cells were subjected to labeling with [U‐^13^C]‐glucose and [1‐^13^C]‐PA, respectively, for 48 h. The labeling extents of FFA (16:0) were measured using LC‐MS‐based metabolomics analysis. Consistently, we observed a significantly higher rate of biosynthesis and lower rate of uptake of FFA (16:0) in human kidney epithelial cells (293T) compared to lung epithelial cells (MRC‐5) (Figure 4E). In MRC‐5 cells, 40% of intracellular FFA (16:0) was taken up from the culture medium, significantly higher than that of 293T cells (Figure 4F). Similar results were also obtained using mouse‐derived cell lines, such as the cardiac muscle cell line (HL‐1) and the immortalized hippocampal cell line (HT22). HL‐1 exhibited higher uptake capacity and lower synthesis of FFA (16:0) compared to HT22 cells (Figure 4G,H). Furthermore, to exclude the effects of glycolysis‐related variability on fatty acid labeling, we employed [U‐^13^C]‐acetate as a tracer to elucidate the capacities of fatty acid biosynthesis in cell lines. The results revealed that 293T and HT22 cell lines exhibited higher capacities for fatty acid synthesis compared to MRC‐5 and HL‐1 cell lines (Figure S10, Supporting Information), thereby providing additional validation of our conclusion. In summary, our investigation characterized the metabolic crosstalk of FFAs between the liver and other tissues, revealing differential contributions from synthesis and uptake routes.

### 2.5 Metabolic Homeostasis Alternations in FFAs Across Tissues During Aging

**Figure 5:**
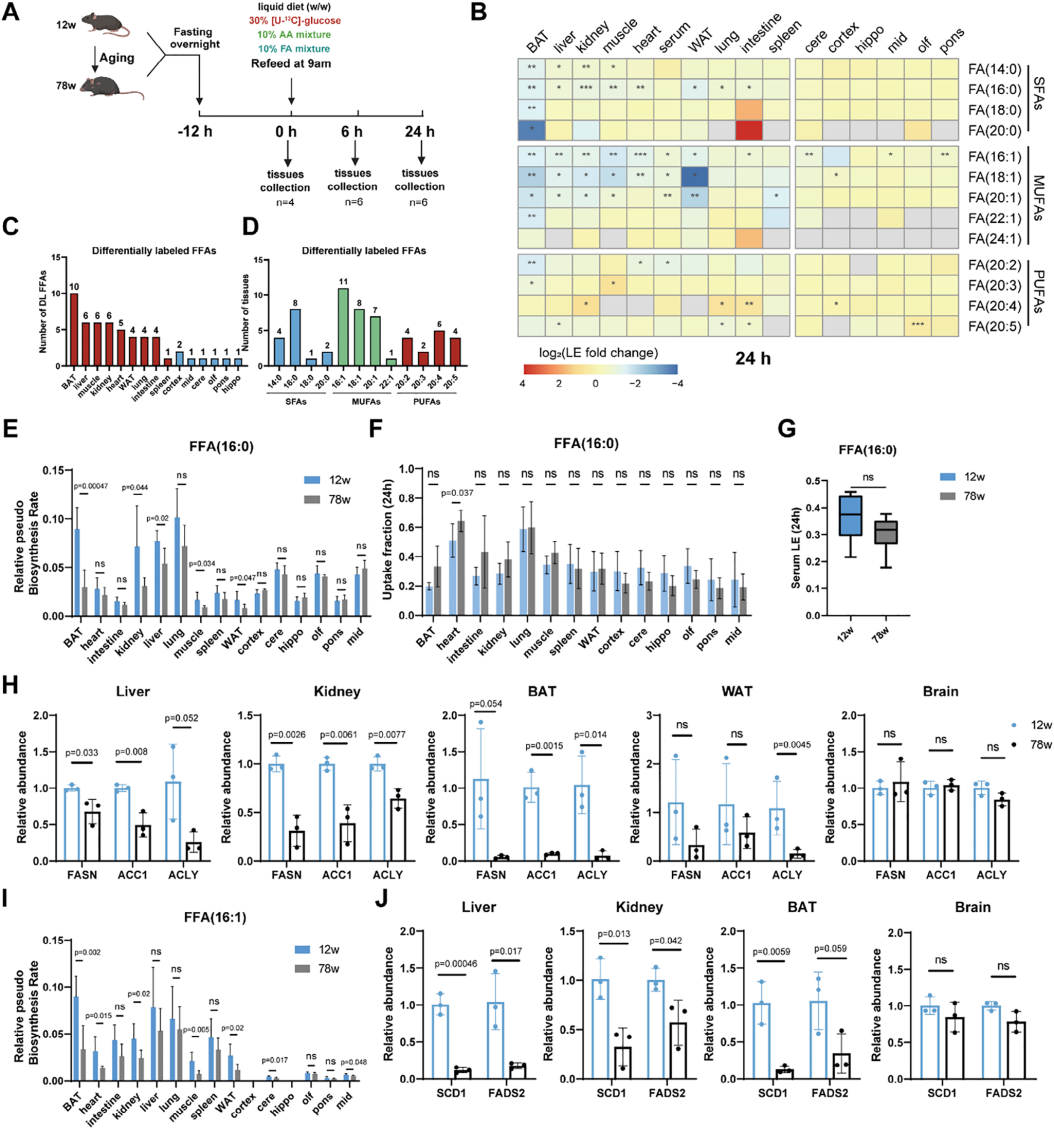
Metabolic alternations in FFAs across tissues during aging. A) Schematic illustration of the in vivo stable‐isotope tracing metabolomics experiment in 12‐week‐old and 78‐week‐old mice (n = 4 for each group at 0 h, n = 6 for each group at 6 and 24 h; C57BL/6J; male). B) A heatmap shows age‐associated alternations in labeling extents of FFAs between 78‐week‐old mice and 12‐week‐old mice after 24 h labeling in multiple tissues. The colors represent the log2 fold change of labeling extents in 78‐week‐old mice compared to 12‐week‐old mice. Gray indicates the value not included in the calculation. C) Statistical analysis of differentially labeled FFAs during aging after 24 h labeling. The bar plot shows the number of differentially labeled FFA in various tissues. Differentially labeled FFAs are defined as significantly changed labeling extents of a specific FFA in aged and young mice (two‐sided Student's t‐test; p value < 0.05). D) The bar plot shows the number of tissues with differentially labeled FFAs. E) Pseudo relative biosynthesis rates of FFA (16:0) after 24 h labeling in mouse tissues. F) Uptake fractions of FFA (16:0) calculated using the model in Figure 4 after 24 h labeling. G) Comparison of serum labeling extents of FFA (16:0) between aging and young mice after 24 h labeling. The centerlines of the boxplots indicate the median values; the lower, and upper lines in boxplots correspond to 25th and 75th quartiles, and the whiskers indicate the largest and lowest values. H) Real‐time PCR analyses of de novo biosynthesis related genes expressions: FASN, ACLY, ACC1 in liver, kidney, BAT, WAT and brain (n = 3 mice for each group; 12‐week‐old and 78‐week‐old; C57BL/6J; male). I) Pseudo relative biosynthesis rates of FFA (16:1) after 24‐h labeling of multiple tissues. J) Real‐time PCR analyses of MUFA biosynthesis related genes expressions: SCD1, FADS2 in liver, kidney, BAT and brain (n = 3 mice for each group;12‐week‐old and 78‐week‐old; C57BL/6J; male). All error bars represent the standard deviation (SD) of mean. All p‐values were calculated by two‐sided Student's t‐test. *p < 0.05, **p < 0.01, ***p < 0.001, and ****p < 0.0001. SFA, saturated fatty acid; MUFA, monounsaturated fatty acid; PUFA, polyunsaturated fatty acid.

The disruption of metabolic flexibility is recognized as a hallmark of aging.^[^ ^13^ ^]^ It remains unclear how systemic changes occur in the adaptation of dietary glucose through FFA biosynthesis during aging. Thus, we further characterized alterations in FFA biosynthesis across multiple tissues during mouse aging. We conducted an in vivo stable‐isotope tracing metabolomics experiment comparing 12‐week‐old and 78‐week‐old mice (n = 6 in each group; **Figure** **5**A). Following 6 or 24 h refeeding, no significant differences were observed in blood glucose concentrations and labeling extent of glucose in the serum between aged and young mice (Figure S11A,B, Supporting Information). Furthermore, tissue‐specific labeling of glucose and pyruvate remained unchanged with age, indicating that glucose uptake and glycolysis—the initial steps of fatty acid synthesis—were not affected during aging (Figure S11C,D, Supporting Information). Subsequently, we compared the labeling extents of 13 FFAs across various mouse tissues, revealing a decline in newly synthesized FFAs in most tissues except brain during aging (Figure 5B). Among the 13 fatty acids analyzed, we observed significant changes in the labeling extent of 10 FFAs in the BAT of 78‐week‐old mice, while only 1–2 FFAs were affected in the brain. This finding suggests that BAT experienced the most significant reduction in FFA synthesis, while fatty acid synthesis in brain remained largely unaffected (Figure 5C). In addition, fatty acid synthesis in the spleen shows no significant alteration, while other peripheral tissues exhibit similar responses to aging (Figure 5C). Examining FFA species, we noted decreases in labeling extents of MUFAs, such as FFA (16:1), FFA (18:1), and FFA (20:1), across most tissues (Figure 5D), indicating a potential role for these fatty acids as crucial mediators of metabolic inflexibility and tissue dysfunction during aging. In contrast, the synthesis of SFAs, such as FFA (16:0), is altered in several tissues, whereas the synthesis of longer‐chain SFAs, such as FFA (18:0) and FFA (20:0), remains largely unaffected. These findings highlight the differential effects of aging on the synthesis of saturated fatty acids of varying chain lengths across different tissues.

Specifically, we observed a decrease in the synthesis rate of FFA (16:0) across multiple tissues during aging, including BAT, white adipose tissue (WAT), kidney, liver, and muscle (Figure 5E). In contrast, the uptake capabilities of FFA (16:0) in most mouse tissues remained unchanged during aging (Figure 5F). Similarly, no alterations were noted in the labeling extents of FFA (16:0) in the circulatory serum between young and old mice (Figure 5G). These findings collectively suggest that the reduced levels of newly synthesized FFA (16:0) may originate from diminished synthesis within tissues rather than disruptions in inter‐tissue crosstalk. To validate our results, we measured the gene expressions of key enzymes involved in FFA (16:0) de novo synthesis, including ATP citrate lyase (ACLY), ACC1 and FASN (Figure S11E, Supporting Information). Consistent with our in vivo tracing metabolomic data, these key enzymes exhibited reduced mRNA expressions in the liver, kidney, BAT, and WAT, while no significant changes were observed in the brain (Figure 5H).

Additionally, MUFAs have been extensively linked to longevity.^[^ ^14^ ^]^ In our data, we observed decreased labeling fractions of MUFAs, such as FFA (16:1), FFA (18:1), and FFA (20:1), in most tissues during aging (Figure 5D). Correspondingly, the biosynthesis rates of MUFAs also tended to decrease in most tissues with aging. Specifically, MUFA biosynthesis in BAT, kidney, and the heart decreased during aging (Figure 5I; Figure S11F,G, Supporting Information). To validate our findings, we measured the mRNA expressions of key enzymes involved in MUFA synthesis, including SCD1 and fatty acid desaturase 2 (FADS2). Consistent with our in vivo tracing metabolomic data, both enzymes exhibited reduced mRNA expressions of SCD1 and FADS2 in the liver, kidney, and BAT, while no significant changes were observed in the brain (Figure 5J). Interestingly, we examined the abundances of FFA (16:0) and MUFAs in young and old mice, which showed no changes during aging (Figure S11H–K, Supporting Information). This observation implies a compensatory mechanism from alternative pathways to counteract the decline in fatty acid biosynthesis, such as heightened lipolysis or reduced fatty acid oxidation. We assessed the expression of key genes involved in lipolysis (*Lipe, Pnpla2*) and fatty acid oxidation (*Cpt1a*) in adipose tissue, liver, and kidney. The results revealed upregulation of lipolytic genes specifically in WAT, with no significant changes observed in the liver or kidney (Figure S11L–N, Supporting Information). Additionally, gene of fatty acid oxidation was downregulated in liver and kidney (Figure S11L–N, Supporting Information). These findings support the notion that increased adipose lipolysis and reduced oxidation may contribute to maintaining fatty acid levels during aging. These results also demonstrate that young mice are capable of rapidly upregulating fatty acid synthesis during the postprandial state. In contrast, aged mice exhibit diminished fatty acid synthesis in peripheral tissues but not in the brain, particularly affecting the synthesis of FFA (16:0) and MUFAs.

## 3 Conclusion and Discussion

Fatty acid biosynthesis plays a pivotal role in the metabolic adaption from fasting to feeding, and maintains metabolic homeostasis and organismal functions in mammals.^[^ ^1^, ^2^, ^3^, ^4^ ^]^ However, tissue‐specific activity for fatty acid biosynthesis and inter‐tissue crosstalk of newly synthesized fatty acids in whole body remain unclear. Here, we utilized an in vivo stable‐isotope tracing metabolomics approach for system‐wide and quantitative analysis of fatty acid biosynthesis and inter‐tissue crosstalk in mice. The results shed light on the intricate interplay between dietary glucose utilization, tissue‐specific fatty acid biosynthesis, and the maintenance of metabolic homeostasis.

Stable‐isotope tracing metabolomics is commonly used to investigate the activity of fatty acid metabolism. Several studies have already conducted calculations of in vivo fatty acid synthesis flux in mouse tissues, but only for a limited number of fatty acids, such as PA and stearic acid, and limited tissues.^[^ ^15^ ^]^ Here, we utilized FAMetA^[^ ^11^ ^]^ to calculate the pseudo relative biosynthesis rates of 13 fatty acids in 15 different tissues, including synthesis, elongation rates, and desaturation rates. We found that newly synthesized fatty acid accumulation in adipose tissue peaks at 6 h postprandially and remains relatively stable between 6 and 24 h (Figure 1D,E; Figure S3, Supporting Information), suggesting a potential short‐term buffering role of adipose tissue in response to dietary intake. In addition to traditional fatty acid synthesis tissues such as liver and adipose tissues, postprandial fatty acid synthesis in the kidney is also highly active (Figure 2B). Previous studies have reported that dysregulation of fatty acid synthesis and uptake in the kidney are associated with chronic kidney disease (CDK),^[^ ^16^ ^]^ highlighting the importance of fatty acid metabolism in the kidney. Additionally, we observed a distinct biosynthetic pattern of FFAs in the brain compared to peripheral tissues. The brain does not tend to utilize glucose for fatty acid synthesis except for PUFAs and PA, while the opposite trend was observed in peripheral tissues. This finding is especially intriguing given the diverse roles of PA in the central nervous system. Previous studies have shown that PA contributes to neuronal function through protein palmitoylation and participates in the hypothalamic regulation of energy homeostasis.^[^ ^17^ ^]^ In the context of aging‐related neurodegenerative diseases, PA has been reported to influence key pathological processes such as tau hyperphosphorylation and amyloid‐β formation.^[^ ^18^ ^]^ These findings suggest that brain‐derived PA may have functional relevance to both normal and pathological brain. Future investigations combining brain specific PA synthesis with behavioral or cognitive analyses may provide further insight into the physiological roles of locally synthesized PA. Interestingly, different brain regions also exhibit heterogeneity in fatty acid synthesis. These results emphasize the tissue‐specific heterogeneity of fatty acid synthesis, which exhibits dynamic spatial and temporal patterns during the postprandial period. However, it should be noticed that given current methodological limitations, pseudo‐biosynthetic rates reflect net fatty acid synthesis at the whole‐cell level and cannot resolve contributions from specific organelles, such as lipid droplets, potentially leading to an overestimation of fatty acid synthesis.

The inter‐tissue metabolic crosstalk of fatty acids plays a crucial role in maintaining overall metabolic homeostasis in the body. Previous studies have reported that oleic acid can regulate fatty acid uptake and oxidation in multiple tissues.^[^ ^2b^ ^]^ Moreover, FFA (16:1) derived from adipose tissue can enhance insulin sensitivity in liver and muscle tissues.^[^ ^19^ ^]^ A major challenge of in vivo stable‐isotope tracing metabolomics is to elucidate the inter‐tissue transport of newly synthesized FFAs. By analyzing the MID of FFAs, we found that four major newly synthesized fatty acids (FFA (14:0), FFA (16:0), FFA (16:1), and FFA (18:1)) in the circulation are mainly derived from the liver (Figure 3), whereas newly synthesized PUFAs in serum may be provided by multiple tissues. These findings broaden the understanding of distinct inter‐tissue metabolic crosstalk of different fatty acids. Additionally, refeeding can attenuate adipocyte lipolysis, primarily through the potent antilipolytic actions of insulin,^[^ ^20^ ^]^ illustrating the shift of FFAs supply in serum from adipose tissue to liver during fasting‐refeeding period. However, it is worth noting that the current dataset allows us to infer potential tissue contributors to circulating FFAs based on labeling dynamics and further direct analysis such as spatial lipidomics using stable‐isotope tracing should be performed to strengthen the findings. Furthermore, through deconvolution of MID of PA and stearic acid, we quantified the crosstalk of PA and stearic acid between the liver and other tissues (Figure 4). The results indicate that the liver, as a central metabolic organ, influences the energy demands and functions of various tissues through fatty acid synthesis and efflux, particularly the lung. Conversely, the majority of newly synthesized FFA (16:0) in the BAT and intestine is produced in situ. The tissue‐specific uptake of fatty acids may be influenced by several factors, including the tissue's ability to uptake FFAs from the bloodstream, its energy requirements, and its role in buffering against dietary glucose. Following refeeding, the intestine and adipose tissue may convert excess glucose into fatty acids for storage, whereas energy‐demanding organs like the heart and lung may rely more on the uptake of FFAs from exogenous sources for energy supply. Collectively, our study provided an analytical framework for systematically exploring inter‐tissue crosstalk of free fatty acids in mammals. It is noteworthy that, although our study focused on the synthesis and uptake of fatty acids across various tissues, we did not investigate the underlying mechanisms of fatty acid transport or differentiate the contributions of distinct transport pathways, such as lipid recycling and the transport of fatty acids in lipid form. Future research should aim to further elucidate the specific mechanisms of inter‐tissue fatty acid crosstalk, as this will enhance our understanding of the processes and significance of fatty acid communication among tissues.

The disruption of metabolic flexibility and nutrient sensing is recognized as hallmarks of aging.^[^ ^13^ ^]^ Fatty acid synthesis has an important role in the aging process.^[^ ^21^ ^]^ In our study, we observed a systemic decline in fatty acid synthesis during aging in peripheral tissues but not in brain, particularly in PA and MUFAs under fasting/refeeding conditions (Figure 5). Further analysis revealed that the inter‐tissue crosstalk of PA remained largely unchanged during aging, and the decrease in newly synthesized PA was attributed to the decline in de novo synthesis within tissues. Additionally, we validated that under fasting conditions, genes related to de novo synthesis were downregulated in aged mice (Figure 5). However, some previous studies indicated the increased expression of FASN in senescent cells and aging mouse livers under non‐fasting conditions.^[^ ^22^ ^]^ Moreover, it has been reported that the capacity to recover serum FFA concentrations to control levels in response to refeeding after short‐term starvation is impaired in aging mammals.^[^ ^23^ ^]^ Consequently, this impairment could potentially underlie the observed decline in FASN activity within the liver during aging, particularly under fasting/refeeding conditions. Furthermore, in alignment with our findings, previous studies proved that the activity of FASN and fatty acid synthesis decreased during aging in adipose tissues.^[^ ^24^ ^]^ Although our data demonstrate an age‐associated decline in fatty acid biosynthesis that correlates with reduced expression of key lipogenic enzymes such as ACLY and FASN, establishing a causal link remains challenging. Previous studies have shown that genetic deletion or pharmacological inhibition of these enzymes leads to reduced palmitate levels and lower circulating lipid concentrations, highlighting their essential roles in lipid synthesis.^[^ ^25^ ^]^ These findings support our observations and underscore the mechanistic relevance of ACLY and FASN in regulating fatty acid metabolism. Nevertheless, direct manipulation of these pathways in aging models—such as through tissue‐specific knockouts or pharmacological interventions—is further required to confirm their roles in age‐related metabolic remodeling.

Previous studies demonstrated that MUFA synthesis is associated with longevity.^[^ ^14^ ^]^ Inactivation of SCD1 suppressed the longevity of *Caenorhabditis elegans* with insulin/IGF‐1 receptor deficiency,^[^ ^26^ ^]^ while SCD1 overexpression prolonged lifespan by inducing MUFA accumulation.^[^ ^14a^ ^]^ Moreover, supplementation of MUFAs is correlated with longevity in humans^[^ ^27^ ^]^ and promotes longevity in rodents^[^ ^28^ ^]^ and C. elegans.^[^ ^14a,c^ ^]^ Furthermore, a recent study detected a decrease in phospholipids and glycerides containing MUFAs.^[^ ^29^ ^]^ In our study, we indicated MUFA synthesis was severely affected in most tissues (Figure 5; Figure S11, Supporting Information). Additionally, we validated that genes related to MUFA synthesis were downregulated in aged mice which are consistent with previous studies in human fibroblasts^[^ ^30^ ^]^ and adipose tissues.^[^ ^31^ ^]^ These results indicated a system‐wide decline of MUFAs synthesis in mice. Interestingly, the intensities of MUFAs remained unchanged during aging, suggesting potential increased lipolysis, reduced oxidation or contributions from other non‐labeled carbon sources in aging mice. Moreover, previous studies reported administration of the pro‐longevity drug rapamycin in mammals increases lipolysis and FFA oxidation.^[^ ^32^ ^]^ Overall, further investigation is warranted to elucidate these possibilities.

Finally, here are some limitations associated with our current study. For example, numerous studies have demonstrated that sex significantly influences glucose and fatty acid metabolism, particularly during aging.^[^ ^29^ ^]^ In females, lipid metabolism is tightly regulated by estrogen, which introduces additional complexity and variability throughout the aging process.^[^ ^33^ ^]^ Therefore, it is important to note that our study primarily focused on male mice and the findings predominantly reflect metabolic adaptations specific to this sex and may not be fully generalizable to females. Comprehensive analysis of both male and female mice would further strengthen our understanding of sex‐dependent FFA metabolism. Additionally, our analysis in this study lacks accurate structural characterization for unsaturated fatty acids. Fatty acids with different double bond positions often exhibit distinct physiological functions; for instance, FFA (20:4) n‐6 acts as a pro‐inflammatory mediator, while FFA (20:4) n‐3 serves as a precursor to FFA (20:5) n‐3, which has anti‐inflammatory properties. Therefore, a careful characterization and quantification of fatty acids with defined double‐bond positions would facilitate a deeper investigation into fatty acid synthesis and inter‐tissue metabolic exchange in mice. Moreover, we did not measure the absolute concentration of FFAs, but rather the intensities of the isotopologues for analysis. However, the labeling extents provided by the in vivo stable‐isotope metabolomics are affected by the pool size. Even if we use the pool size correction to generate the relative biosynthesis rate of FFAs (Figure 2), we still need to consider the bias caused by the entire method system. Furthermore, since we used diet to perform in vivo stable‐isotope tracing in mice, although invasive labeling methods were avoided, there may be individual differences in mouse absorption capacity, which in turn led to differences in labeling efficiency. Although there were no significant differences in food intake among the mice (Figure S12, Supporting Information), and the variation in glucose labeling in mouse serum after 24 h of labeling was minimal (Figure S11B, Supporting Information), these limitations should be taken into account when interpreting the results.

## 4 Experimental Section

### Chemicals

LC–MS grade water (H_2_O) was purchased from Honeywell (Muskegon, MI, USA). LC–MS grade acetonitrile (ACN) was purchased from Merck (Darmstadt, Germany). Ammonium hydroxide (NH_4_OH) and ammonium acetate (NH_4_OAc) were purchased from Sigma (St. Louis, MO, USA). Stable‐isotope tracing reagents including [U‐^13^C]‐glucose, [1‐^13^C]‐palmitic acid, and [U‐^13^C]‐acetate were purchased from Cambridge Isotope laboratories (MA, USA).

### Stable‐Isotope Tracing Experiments in Cells

293T and HT22 were cultured in Dulbecco Modified Eagle's Medium (DMEM) with 10% dialyzed fetal bovine serum (dFBS) and 1% penicillin/streptomycin (PS). MRC‐5 and HL‐1 were cultured in Minimum Essential Medium (MEM) with 10% dialyzed fetal bovine serum (dFBS) and 1% penicillin/streptomycin (PS). When cells reached 80% confluence, the unlabeled medium was aspirated, and cells were washed with PBS before being changed to fresh medium. For [U‐^13^C]‐glucose tracing experiment, the cell medium was changed to glucose‐free DMEM fresh medium containing 25 mm [U‐^13^C]‐glucose, 10% dFBS, and 1% PS for 293T and HT‐22, and glucose‐free MEM fresh medium containing 5.15 mm [U‐^13^C]‐glucose, 10% dFBS, and 1% PS for MRC‐5 and HL‐1. For [1‐^13^C]‐palmitic acid tracing experiments, [1‐^13^C]‐palmitic acid was first dissolved in Isopropyl alcohol to make 300 mm stock solution. The stock solution was diluted with DMEM containing 10% FBS, 1% PS, and 1% bovine serum albumin (BSA) for 293T and HT22 cells or MEM for MRC‐5 and HL‐1 cells to a final concentration of 0.1 mm. For [U‐^13^C]‐acetate tracing experiment, the cell medium was changed to DMEM fresh medium containing 2.5 mm [U‐^13^C]‐acetate, 10% dFBS, and 1% PS.

After 48 h of labeling, the culture medium was quickly aspirated, and cells were washed twice with PBS. The cell plates were swiftly placed on dry ice and rapidly quenched by adding 1 mL of pre‐cooled MeOH:ACN:H_2_O (2:2:1, v/v/v) extraction solution. The plates were then incubated at ‐80 °C for 40 min. The cells were scraped from the plate and transferred to a centrifuge tube, along with an additional 500 µL of extraction solution collected from washing the dish. The samples were then vortexed for 1 minute, followed by centrifugation at 16200 x g for 15 min at 4 °C. The supernatant was collected and evaporated to dryness in a vacuum concentrator. The dried extracts were kept in −80 °C. Before LC–MS analysis, the dried extracts were reconstituted in 100 µL of ACN:H_2_O (1:1, v/v), subjected to 10 min of sonication (50 Hz, 4 °C), and then centrifuged at 16200 x g for 15 min at 4 °C to remove insoluble material. Finally, the supernatants were transferred to HPLC glass vials for LC‐MS analysis.

### In Vivo Stable‐Isotope Tracing Experiments in Mice

12‐week‐old male mice and 78‐week‐old male mice (C57BL/6J) were group‐housed in a barrier facility at room temperature of 22 °C with 50% humidity and 12 h light/12 h dark cycles. All mice were fed with liquid diet containing 30:10:10% (w/v, glucose: soy protein: coconut milk) for one week prior to the in‐vivo stable‐isotope tracing experiment. There were no significant differences in food intake among the 8 mouse cages (Figure S12, Supporting Information). Subsequently, the mice were subjected to an overnight fast and re‐fed with liquid diet containing 30:10:10% (w/v, [U‐^13^C]‐glucose: soy protein: coconut milk) in the following morning at 9 a.m. Whole blood of mice was collected via eye enucleation after 6 h and again after 24 h of refeeding. The mice were then immediately sacrificed by cervical dislocation. BAT, heart, intestine, kidney, liver, lung, muscle, spleen, WAT, cortex, hippocampus, midbrain, pons, cerebellum and olfactory bulb were quickly dissected, immediately frozen in liquid nitrogen and stored at ‐80 °C until metabolite extraction. The whole blood was centrifuged at 3000 x g for 30 min at room temperature and the supernatant was collected to obtain serum. There were 6 mice at each time point (6 and 24 h of refeeding) in two aged groups (12‐week and 78‐week; n = 24 in total). For controls, samples from mice at the end of fasting (0 h) were also collected in two aged groups (12‐week and 78‐week; n = 4 in each group, and n = 8 in total).

For metabolite extraction, the frozen mouse tissues were transferred into homogenizer tubes and homogenized with H_2_O at a ratio of 200 µL H_2_O per 20 mg tissue and ceramic beads using a homogenizer (JXFSTPRP‐CL, Shanghai Jingxin Experimental Technology) at the low‐temperature condition. Two hundred microliters homogenized solution was taken out for each sample and 800 µL extraction solution (ACN: MeOH = 1:1, v/v) was added for metabolite extraction. The mixture solution was vortexed for 30 s, and sonicated for 10 min at 4 °C water bath. Then the sample was incubated for 1 h at −20 °C, followed by centrifugation for 15 min at 16200 x g and 4 °C. The supernatant was taken to a new 1.5‐mL EP tube and evaporated to dryness at 4 °C in a vacuum concentrator. The following extraction processes were the same as those for cell samples. The recovery rates for FFA extraction were shown in Figure S13 (Supporting Information).

### LC‐MS Analysis

Metabolomics data of cell and mice samples were acquired using a Vanquish UHPLC coupled to an Orbitrap Exploris 480 (ThermoFisher Scientific, United States) with Xcalibur software (version 4.4.16.14, Thermo Fisher Scientific, USA). Phenomenex Kinetex C18 column (particle size, 2.6 µm; 100 mm (length) × 2.1 mm (i.d.)) was used for LC separation of FFAs and the column temperature was kept at 25 °C. The injection volume was 2 µL. The injection volume was 2 µL. Mobile phases, linear gradient eluted and ESI source parameters followed previous publication.^[^ ^34^ ^]^

### Data Processing

The stable‐isotope tracing metabolomics data analysis followed the previous publication.^[^ ^34^, ^35^ ^]^ Each tissue was processed individually. The raw data of all samples first (raw) was converted to.mzXML (for full scan mode) and.mgf (for ddMS2 mode) format using ProteoWizard (version 3.0.20360). Then, the mzXML data files of unlabeled samples were grouped for peak detection and alignment using R package “xcms” (version 3.12.0). Free fatty acids were identified and confirmed using chemical standards by matching MS1, retention time and MS2 spectra (Table S1, Supporting Information). Then, the annotation table, a previously generated xcmsSet file, unlabeled data (mzXML), and labeled data (mzXML) were input into the published R package “MetTracer” for extraction of labeled isotopologues of each FFA.^[^ ^34^ ^]^ Next, natural isotope correction was performed using the R package “AccuCor” (version 0.2.4; https://github.com/XiaoyangSu/AccuCor). The parameters for MetTracer were set as follows: rt.extend, 15; value, “maxo”; equipment, “Orbitrap”; ppm, 10; res.define, 200; resolution, 60 000; d.extract, “labelled”; correct.iso, “TRUE”; adj.contaminate, “TRUE”. The output of MetTracer were corrected intensities for each isotopologue of FFAs and their MID, shown in Tables S2 and S3 (Supporting Information). The labeled fraction was defined as the ratio of the intensity of each isotopologue for one FFA to the total intensity of that specific FFA. Therefore, MID was further defined as the labeled fractions of all isotopologues for a specific FFA. In the labeled samples, if the labeled fraction of one isotopologue (except M0) for a specific FFA was larger than 0.005 in more than 50% of samples, this FFA was considered to be isotopically labeled.

### Glucose Concentration Measurements

Glucose concentration was measured using Glucose assay kit (O‐toluidine method, Beyotime, S0201S). Five microliters of standard solution or serum sample was transferred to a PCR tube, then 185 µL of Glucose Assay Reagent containing the o‐toluidine was added into tube. After vortex mixing, the mixture was centrifuged at 5000×g for several seconds to settle the liquid at the bottom of the tube. The sample was then heated at 95 °C for 8 min, and cooled to 4 °C. The absorbance of reaction liquid at 630 nm was measured, and then used to calculate the glucose concentration in the sample based on the standard curve.

### Quantitative Real‐Time PCR

The tissue was ground into powder in liquid nitrogen. Ten to twenty milligrams of tissue was used for RNA extraction. The RNA was isolated using RNA Easy Fast Tissue Kit (TIANGEN, DP451) in accordance with manufacturer's instruction. The quantity of the extracted RNA was determined using NanoDrop (ThermoFisher Scientific, United States). First‐strand cDNA was synthesized from 1 µg of RNA using Reverse Transcriptase M‐MLV (RNase H‐) (Takara, Japan). Real‐time PCR analysis was performed using the QuantStudio 6 Flex real‐time PCR system with 2x SYBR Green qPCR Master Mix (SelleckChem). The 2 ^−ΔΔCT^ method was employed to calculate the gene expression data, with the actin gene serving as the reference control.

### Intensity of ^13^C‐Labeled FFA, Labeling Extent, ^13^C Enrichment Calculation

The intensity of ^13^C‐labeled FFA (*I*_13*C* − *FFA*_) was represented as the incorporation of ^13^C atoms from dietary [U‐^13^C]‐glucose into the newly synthesized FFA, calculated by Equation (1):

(1) $$I_{13C-FFA}=\sum_{i=1}^{C}i\times I_{Mi}$$

where *I_Mi_* is the peak intensity of isotopologue *Mi*, and *C* is the total carbon number of the fatty acid.

Labeling extent (LE) and ^13^C enrichment denote the degrees of labeled fractions at the molecular level and the carbon atom level for a specific fatty acid, respectively. Labeling extent and ^13^C enrichment were calculated by Equations (2) and (3):

(2) $$LE=\frac{\sum_{i=1}^{C}I_{Mi}}{\sum_{i=0}^{C}I_{Mi}}=1-L_{M0}$$

(3)

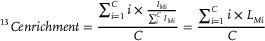

where *L_Mi_* is the labeled fraction of isotopologue *Mi*, and C is the total carbon number of the fatty acid. The calculation results are provided in Table S4 (Supporting Information).

### Pseudo Relative Biosynthesis Rate Determination of FFAs

A previously published R package FAMetA,^[^ ^11^ ^]^ was used to calculate FFA synthesis flux, g(t), which represents the fraction of newly synthesized FFAs during labeling time t. Specifically, MID of each FFA measured by LC‐MS was inputted into FAMetA. FAMetA employs a quasi‐multinomial model to fit the theoretical MIDs of FFAs to their experimental MIDs. This is achieved by iteratively adjusting model parameters, including the labeling fractions of precursor acetyl‐CoA isotopologues (D2, D1, and D0) and synthesis flux parameters such as synthesis (S), elongation (E), and desaturation (Δ). For each fatty acid, FAMetA only outputs one synthesis flux parameter based on the specific FFA synthesis route, which is uniformly referred to as “g(t)” in this work (Table S5, Supporting Information). Due to the dependence of g(t) values on the pool size of FFAs, it was next corrected for the FFA pool size. Intensities of FFAs in all tissues were rescaled to [0,1] to generate the pool size factors. Then pseudo relative biosynthesis rate of each FFA was calculated through multiplying g(t) by the pool size factor for each tissue (Table S5, Supporting Information).

### Mass Isotopologue Distribution Similarity Score Calculation

MID similarity score was calculated by normalized Manhattan distance as in Equation (4):

(4) $$\mathrm{Similarity}\;\mathrm{score}=1/\left(1+\sum_{i=1}^{C}\left|L_{a,i}-L_{\mathrm{serum},i}\right|\right)$$

where *L*_*a*,*i*_ is the labeled fraction of *M_i_* in tissue a, *L*_*serum*,*i*_ is the labeled fraction of *M_i_* in serum, C is the total carbon number of the FFA. The MID similarity scores of FFAs between tissue and serum were provided in Table S6 (Supporting Information).

### Quantitative Analysis of Inter‐Tissue FFA Crosstalk

The isotopologue pattern of a specific FFA can be decomposed into the fraction of biosynthesis source and the fraction of uptake source, with the latter reflects the FFA metabolic crosstalk between different tissues. For FFA (16:0), the model of deconvolution of de novo synthesis and uptake is characterized by Equations (5) – (7):

(5) $$D_{i}=C_{8}^{m}\times C_{8-m}^{n}\times {x_{2}}^{m}\times {x_{1}}^{n}\times {x_{0}}^{8-m-n}$$

(6) $$U_{i}=L_{Mi,serum}/\sum_{i}^{C}L_{Mi,serum}$$

(7) $$M_{i,pre}=\alpha \times \frac{D_{i}}{\sum_{i=1}^{C}D_{i}}+\beta \times U_{i}$$

where *D_i_* represents theoretical de novo synthesis fraction of isotopologue_*i*_, *U_i_* represents normalized L_*Mi*_ in serum, m represents putative number of acetyl‐coenzyme A with 2 carbons labeled, n represents putative number of acetyl‐coenzyme A with 1 carbon labeled, *x_i_* represents labeled fraction of *M*i of acetyl‐coenzyme A, α represents the fraction of de novo synthesis, β represents the fraction of uptake from serum. The model parameters α, β, *x*_0_, *x*_1_, *x*_2_ are inferred by minimizing the sum of squared errors between observed and fit labeling (M_*i*,*pre*_) with nonlinear optimization within SciPy.^[^ ^36^ ^]^

For FFA (18:0), the model of deconvolution of synthesis and uptake is characterized by Equations (8) and (9):

(8) $$E_{i}=D_{i}\times x_{0}+D_{i-1}\times x_{1}+D_{i-2}\times x_{0}$$

(9) $$M_{i,pre}=\alpha \times \frac{E_{i}}{\sum_{i=1}^{C}E_{i}}+\beta \times U_{i}$$

where *x_i_* represents labeled fraction of *M*i of acetyl‐coenzyme A from the previous result of FFA (16:0). The parameters were inferred by the same approach (Table S7, Supporting Information).

### Statistical Analysis

All bars represented mean ± SD. All statistical analyses were performed using GraphPad Prism (v 9.0), Microsoft Excel 2019, R (version 4.2.3), and PyCharm (version 23.1.4).

### Ethical Statement

The animal experiments were compliant with the ethical guidelines of the Institutional Animal Care and Use Committees of Interdisciplinary Research Center on Biology and Chemistry, Shanghai Institute of Organic Chemistry, Chinese Academy of Sciences (approved project number: ECSIOC_2023‐23).

## Conflict of Interest

The authors declare no conflict of interest.

## Author Contributions

Z.J.Z. and B.X. conceived the idea and designed the experiments. B.X. and R.W. designed and conducted major animal experiments, acquired and processed the metabolomic data. B.X. carried out the biological experiments and data analysis. T.K. and W.L. contributed to samples preparation. Z.J.Z. and B.X. wrote the manuscript. Z.J.Z. supervised the project.

## Supporting information



Supporting Information

Supplemental Table 1

Supplemental Table 2

Supplemental Table 3

Supplemental Table 4

Supplemental Table 5

Supplemental Table 6

Supplemental Table 7

## Data Availability

The data that support the findings of this study are openly available in [National Omics Data Encyclopedia under Project] at [https://www.biosino.org/node/project/detail/OEP005595], reference number [5595].
