# Supplementary material for: Unraveling Tissue‐Specific Fatty Acid Biosynthesis and Inter‐Tissue Crosstalk in Mice through Stable‐Isotope Tracing Metabolomics
Source: Adv Sci (Weinh). 2025 May 30;12(31):e03662. doi: 10.1002/advs.202503662 (PMC12376646; doi:10.1002/advs.202503662)
Supplement: Supplementary file 1 — Supporting Information [file ADVS-12-e03662-s008.docx]

Supporting Information

Unraveling tissue-specific fatty acid biosynthesis and inter-tissue crosstalk in mice through stable-isotope tracing metabolomics

Beizi Xing, Ruohong Wang, Tianzhang Kou, Wenbin Liu, and Zheng-Jiang Zhu^*^

**List of Supplementary Figures**

**Figure S1:** Extracted ion chromatogram (EIC) plots of 21 FFAs in mouse muscle samples.

**Figure S2:** ^13^C enrichment and intensities of labelled FFAs in mice.

**Figure S3:** Different accumulation rates of newly synthesized FFAs in mice.

**Figure S4:** Pseudo relative biosynthesis rates of FFAs in different tissues.

**Figure S5:** Mass isotopologue distributions (MIDs) in tissues and serum.

**Figure S6:** Metabolic crosstalk of FFAs between the circulatory serum and tissues.

**Figure S7:** The observed MIDs and predicted MIDs of FFA (16:0) obtained from deconvolution algorithm.

**Figure S8**: The observed MIDs and predicted MIDs of FFA (18:0) obtained from deconvolution algorithm.

**Figure S9:** Coefficients for the MID deconvolution.

**Figure S10:** Labeling extents of FFA (16:0) in 293T, MRC-5, HL-1 and HT22 cell lines using [U-^13^C]-acetate labeling for 48 h.

**Figure S11:** Metabolic alternations in FFAs across tissues during aging.

**Figure S12:** Food intake of mice during the 6-day experiment.

**Figure S13:** Recovery rates for FFA extraction in serum and liver samples.

**List of Supplementary Data Files**

**Table S1:** FFA annotations of mouse tissues dataset acquired on Orbitrap Exploris 480.

**Table S2:** Isotopologue intensities of FFAs in 12-week-old and 78-week-old mice after 6 h and 24 h labeling.

**Table S3:** MIDs of FFAs in 12-week-old and 78-week-old mice after 6 h and 24 h labeling.

**Table S4:** Intensities of ^13^C-labeled FFAs, labeling extents of FFAs, and ^13^C enrichments of FFAs in 12-week-old and 78-week-old mice after 6 h and 24 h labeling.

**Table S5:** Pseudo relative biosynthesis rates of FFAs in 12-week-old mice after 24 h labeling.

**Table S6:** MID similarities with p values and LE ratios of FFAs between tissues and the circulatory serum**.**

**Table S7:** The uptake fractions, the biosynthesis fractions and predicted MIDs of FFA (16:0) and FFA (18:0) in tissues.


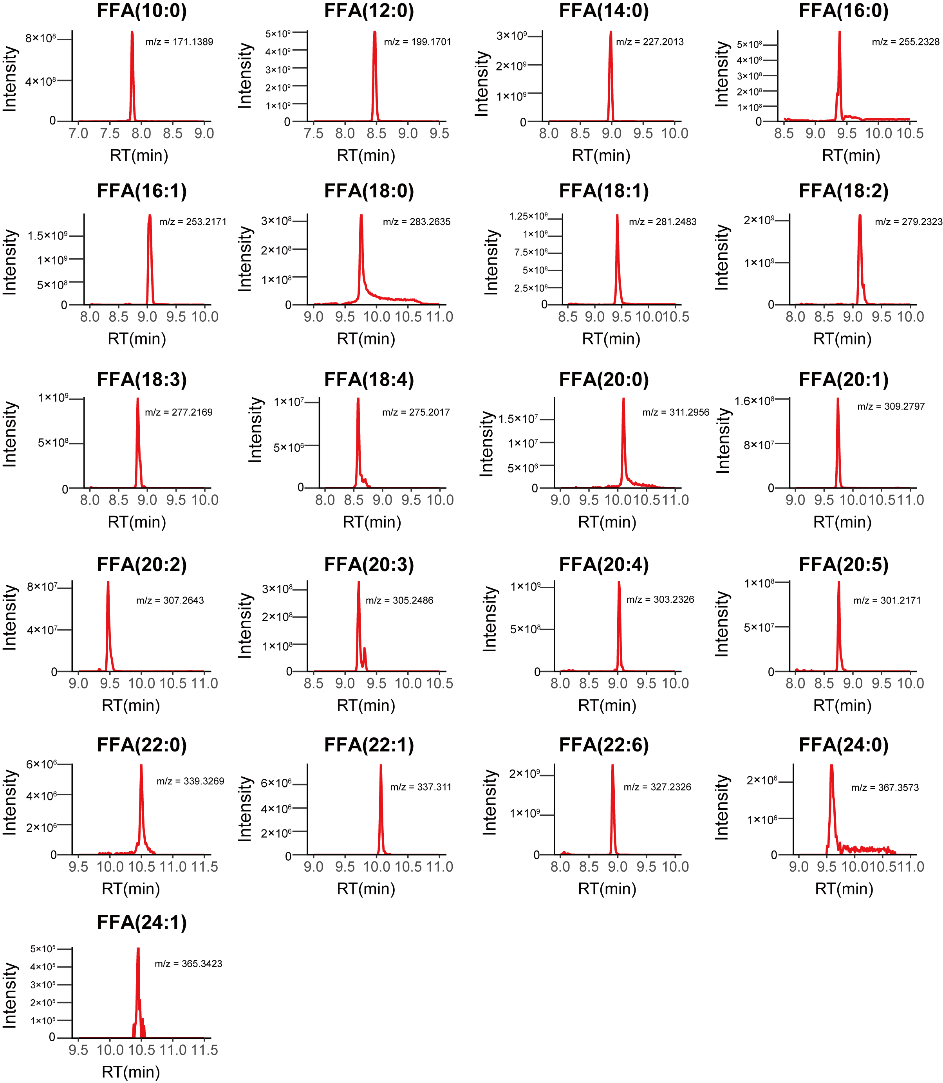


**Figure S1.** Extracted ion chromatogram (EIC) plots of 21 FFAs in mouse muscle samples.


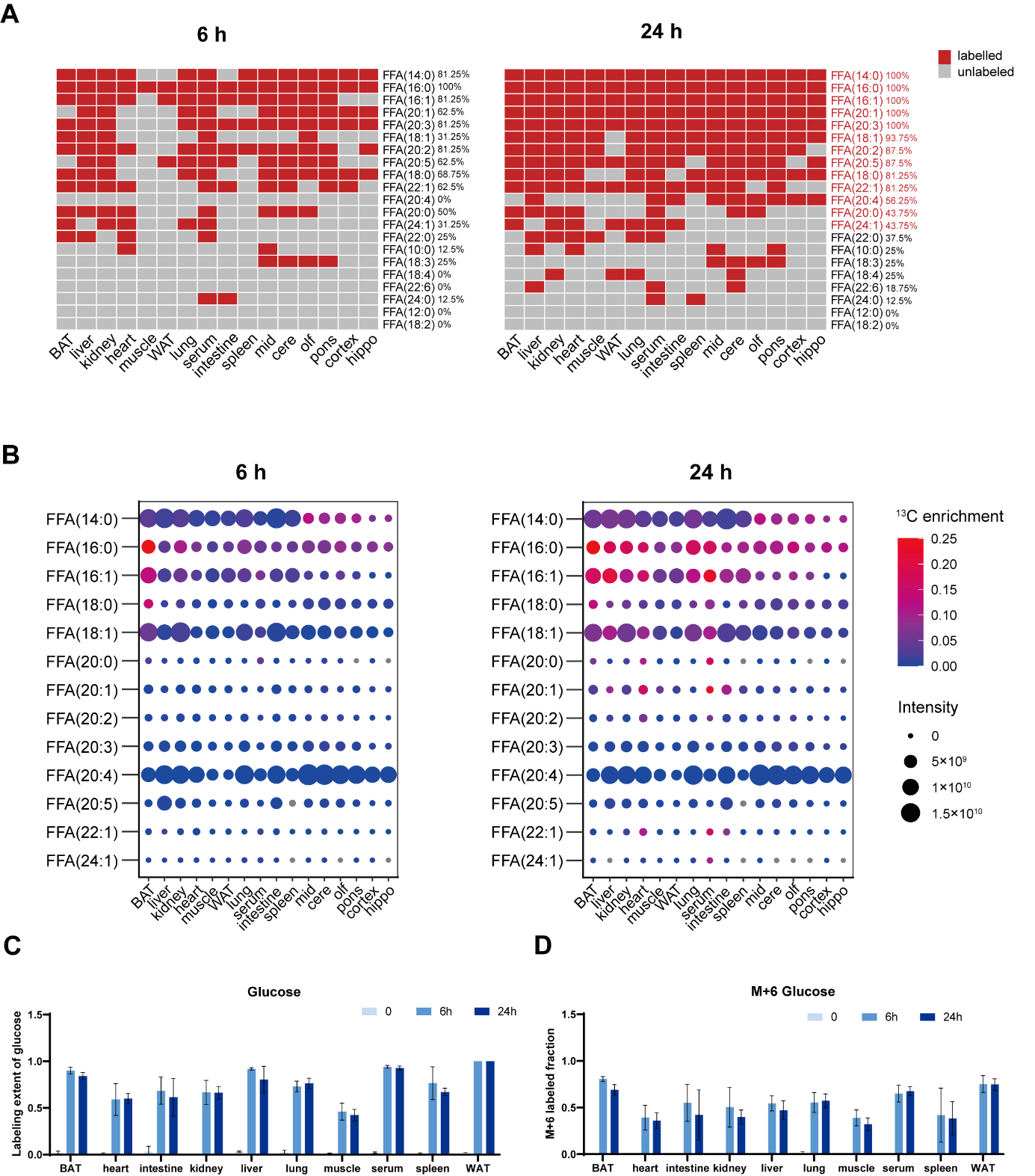


**Figure S2.** ^13^C enrichment and intensities of labelled FFAs in mice. **A**) Heatmaps show labeled and unlabeled FFAs in all tissues after 6 h and 24 h labeling (n=6 at each time point; 12-week-old; C57BL/6J; male). If the labeled fraction of one isotopologue (except M0) for a specific FFA was larger than 0.005 in more than 50% of samples (n=6 mice), the FFA was considered to isotopically labeled. Red and grey represent labelled FFAs and unlabeled FFAs in tissues, respectively. The percentages next to each FFA represent the proportion of tissues in which the FFA was labeled related to the total number of tissues. **B**) Bubble plots show the ^13^C enrichments and intensities of 13 FFAs after 6 h and 24 h labeling. ^13^C enrichment was determined by assessing the molar abundance of ^13^C atoms relative to the total molar abundance of carbon atoms (see Methods, Equation 3). The color and circle size show median value of ^13^C enrichment and intensity in biological samples, respectively (n=6 mice). **C**,**D**) Labeling extents of glucose and labeled fractions of M+6 glucose in tissues after 6 h and 24 h labeling. Error bars represent the standard deviation (SD) of mean (n=6 mice; 12-week-old; C57BL/6J; male).


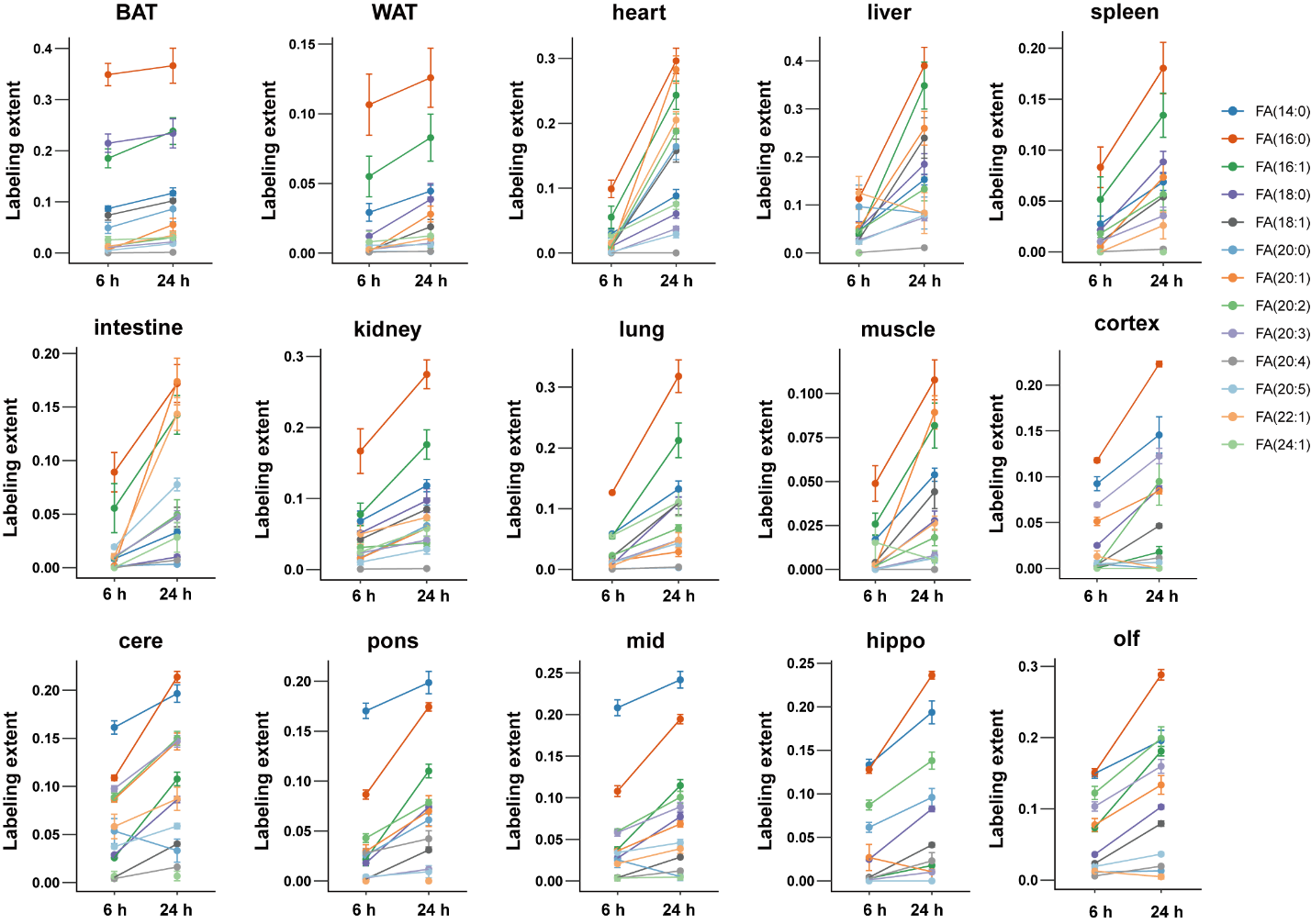


**Figure S3**. Different accumulation rates of newly synthesized FFAs in mice. Line charts show labeling extents of 13 FFAs in various tissues after 6 h and 24 h labeling. Error bars represent the standard deviation (SD) of mean (n=6 at each time point; 12-week-old; C57BL/6J; male). BAT, brown adipose tissue; WAT, white adipose tissue; intestine, small intestine; mid, midbrain; cere, cerebellum; olf, olfactory bulb; hippo, hippocampus.


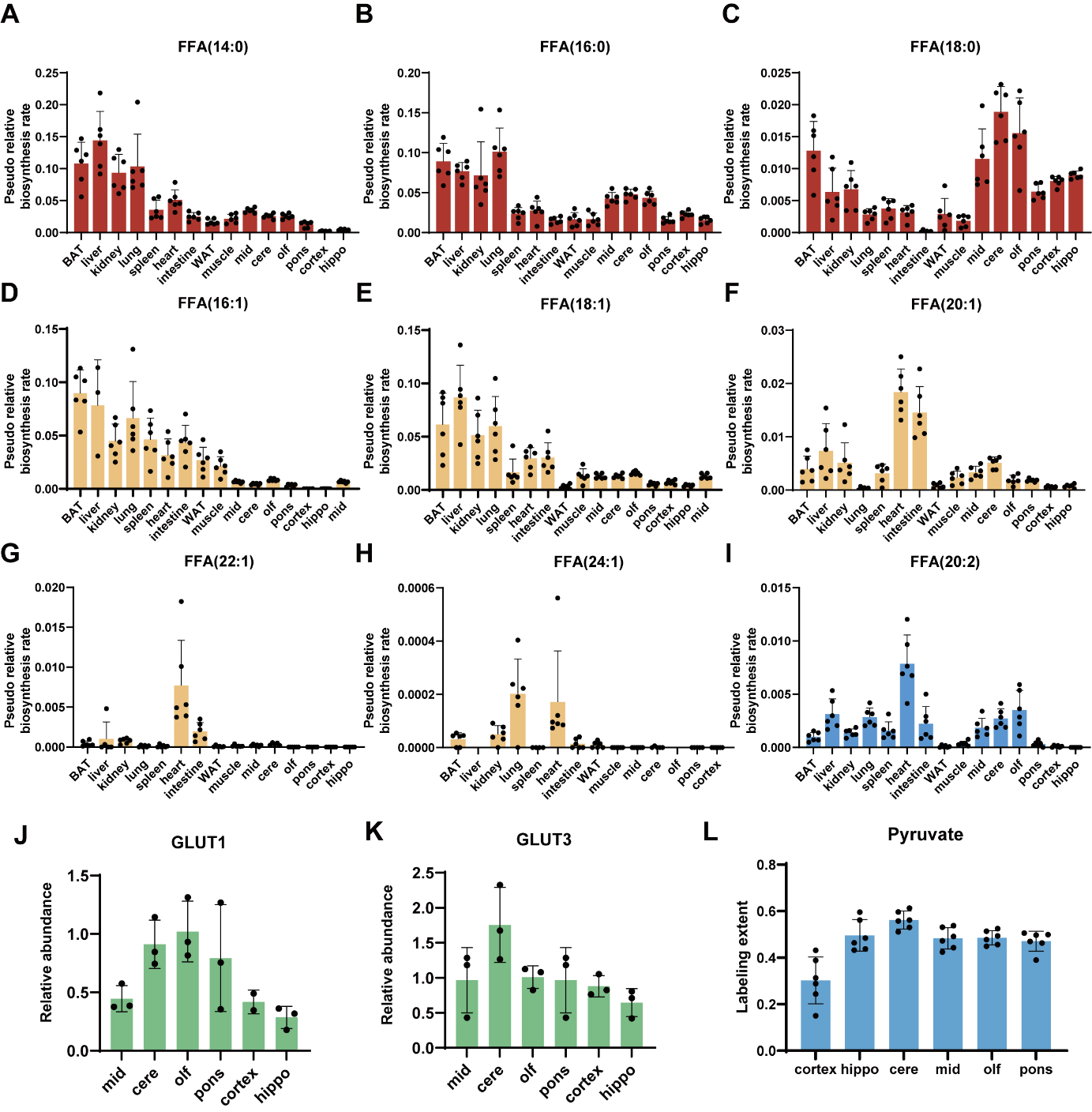


**Figure S4**. Pseudo relative biosynthesis rates of FFAs in different tissues. **A**-**I**) Bar plots show the relative pseudo biosynthesis rates of FFA (14:0), FFA (16:0), FFA (18:0), FFA (16:1), FFA (18:1), FFA (20:1), FFA (22:1), FFA (24:1) and FFA (20:2) in 15 tissues after 24 h labeling. Error bars represent the standard deviation (SD) of mean (n=6 mice; 12-week-old; C57BL/6J; male). **J**,**K**) Gene expression of GLUT1 and GLUT3 in brain regions. Error bars represent the standard deviation (SD) of mean (n=3 mice; 12-week-old; C57BL/6J; male). **L**) Labeling extents of pyruvate in brain regions after 24 h labeling. Error bars represent the standard deviation (SD) of mean (n=6 mice; 12-week-old; C57BL/6J; male).


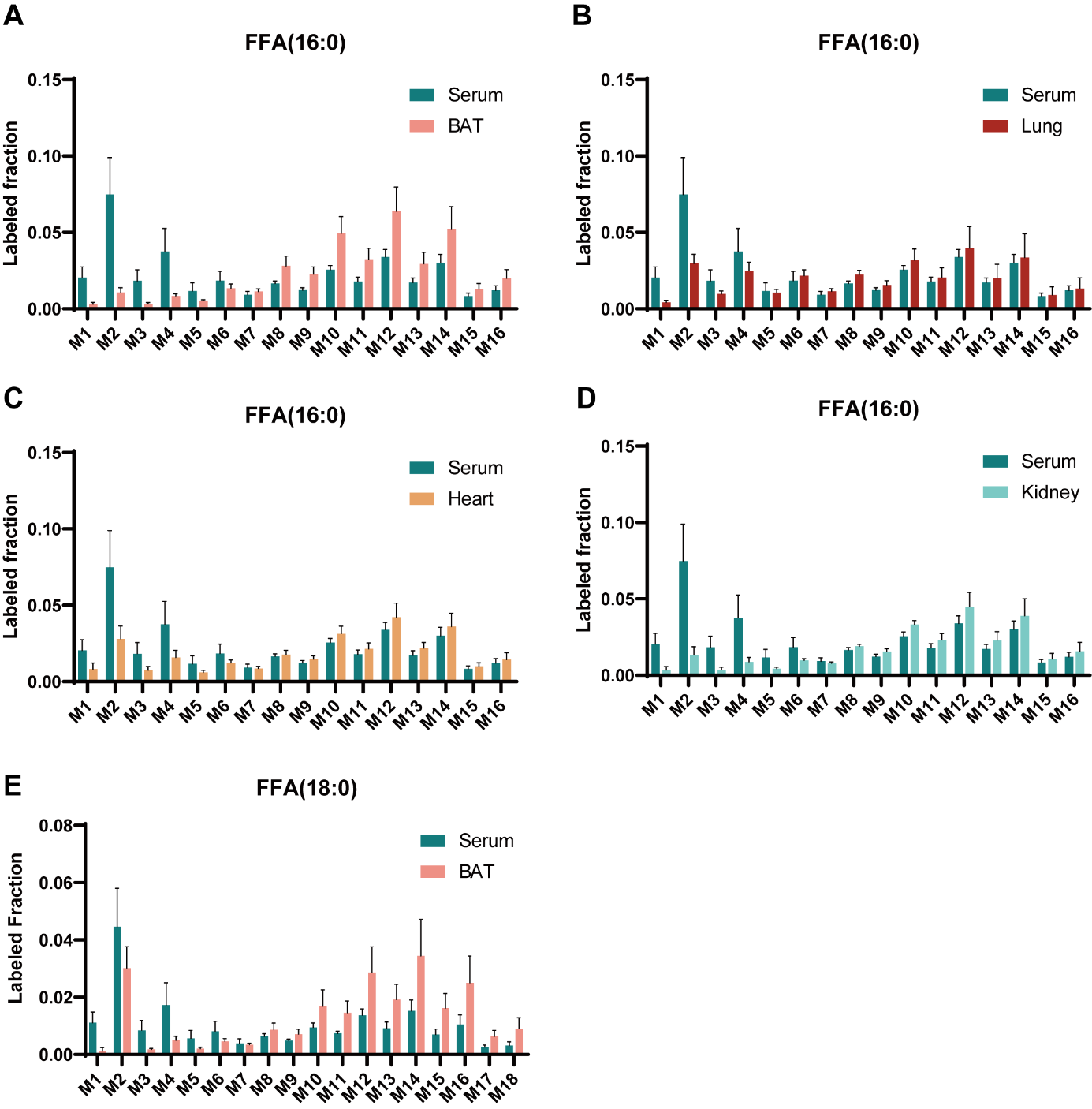


**Figure S5**. Mass isotopologue distributions (MIDs) in tissues and serum. **A**-**D**) Comparisons of MIDs of FFA (16:0) in BAT, lung, heart, and kidney with those in serum after 24 h of labeling. Error bars represent the standard deviation (SD) of mean (n=6 mice; 12-week-old; C57BL/6J; male). **E**) MIDs of FFA (18:0) in BAT and serum after 24 h labeling. Error bars represent the standard deviation (SD) of mean (n=6 mice; 12-week-old; C57BL/6J; male).


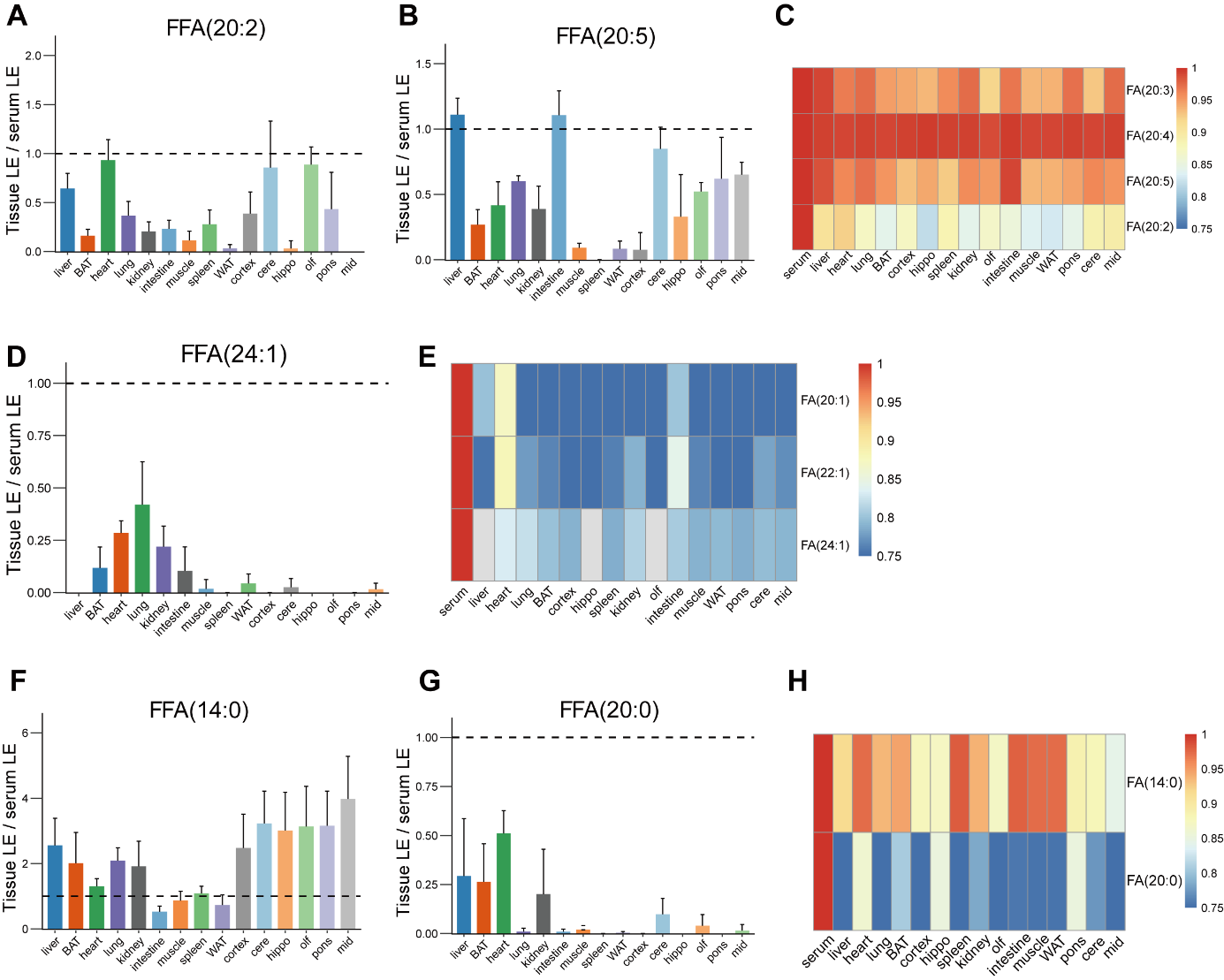


**Figure S6**. Metabolic crosstalk of FFAs between the circulatory serum and tissues. **A**,**B**) Comparisons of labeling extents of FFA (20:2) and FFA (20:5) in different tissues to those in serum after 24 h labeling. Error bars represent the standard deviation (SD) of mean (n=6 mice; 12-week-old; C57BL/6J; male). **C**) A heatmap shows MID similarity scores of FFA (20:2), FFA (20:4), FFA (20:3), FFA (20:5) between each tissue and serum after 24 h labeling. The MID similarity scores were calculated by Equation (4) in Methods. The color represents the median values of MID similarity scores in biological samples (n=6 mice). **D**) Comparisons of labeling extents of FFA (24:1) in different tissues to those in serum after 24 h labeling. Error bars represent the standard deviation (SD) of mean (n=6 mice; 12-week-old; C57BL/6J; male). **E**) A heatmap shows MID similarity scores of FFA (20:1), FFA (22:1) and FFA (24:1) between each tissue and serum after 24 h labeling. The color represents the median values of scores in biological samples (n=6 mice). **F,G**) Comparisons of FFA (14:0) and FFA (20:0) labeling extents between serum and other tissues after 24 h labeling. Error bars represent the standard deviation (SD) of mean (n=6 mice; 12-week-old; C57BL/6J; male). **H**) A heatmap shows MID similarity scores of FFA (14:0) and FFA (20:0) between each tissue and serum after 24 h labeling. The color represents the median values of scores in biological samples (n=6 mice).


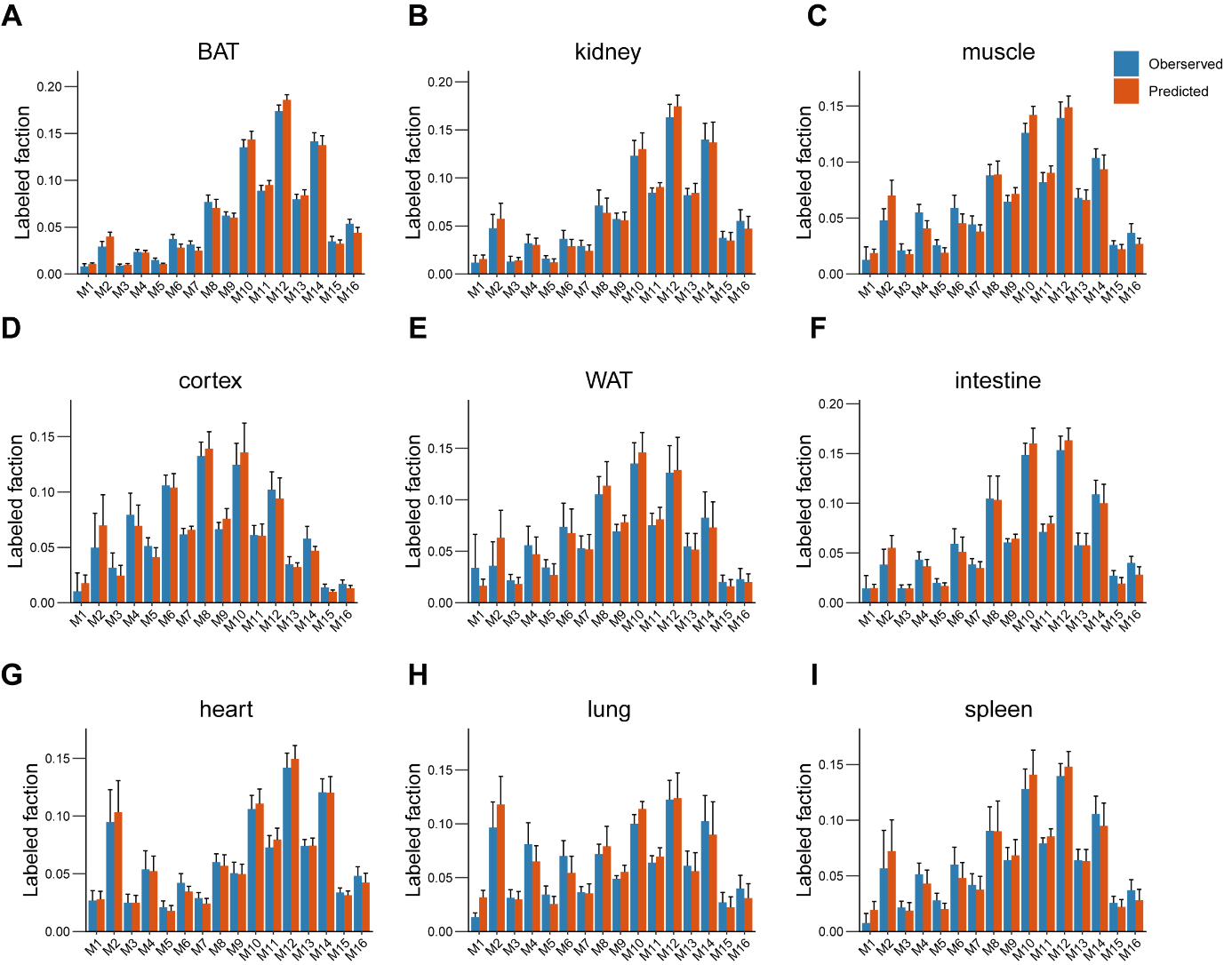


**Figure S7.** The observed MIDs and predicted MIDs of FFA (16:0) obtained from deconvolution algorithm. **A**-**I**) Comparisons of observed MIDs and predicted MIDs of FFA (16:0) obtained from deconvolution algorithm in BAT, kidney, muscle, cortex, WAT, small intestine, heart, lung, spleen. 24 h labeling data were used to input the model. Error bars represent the standard deviation (SD) of mean (n=6 mice; 12-week-old; C57BL/6J; male).


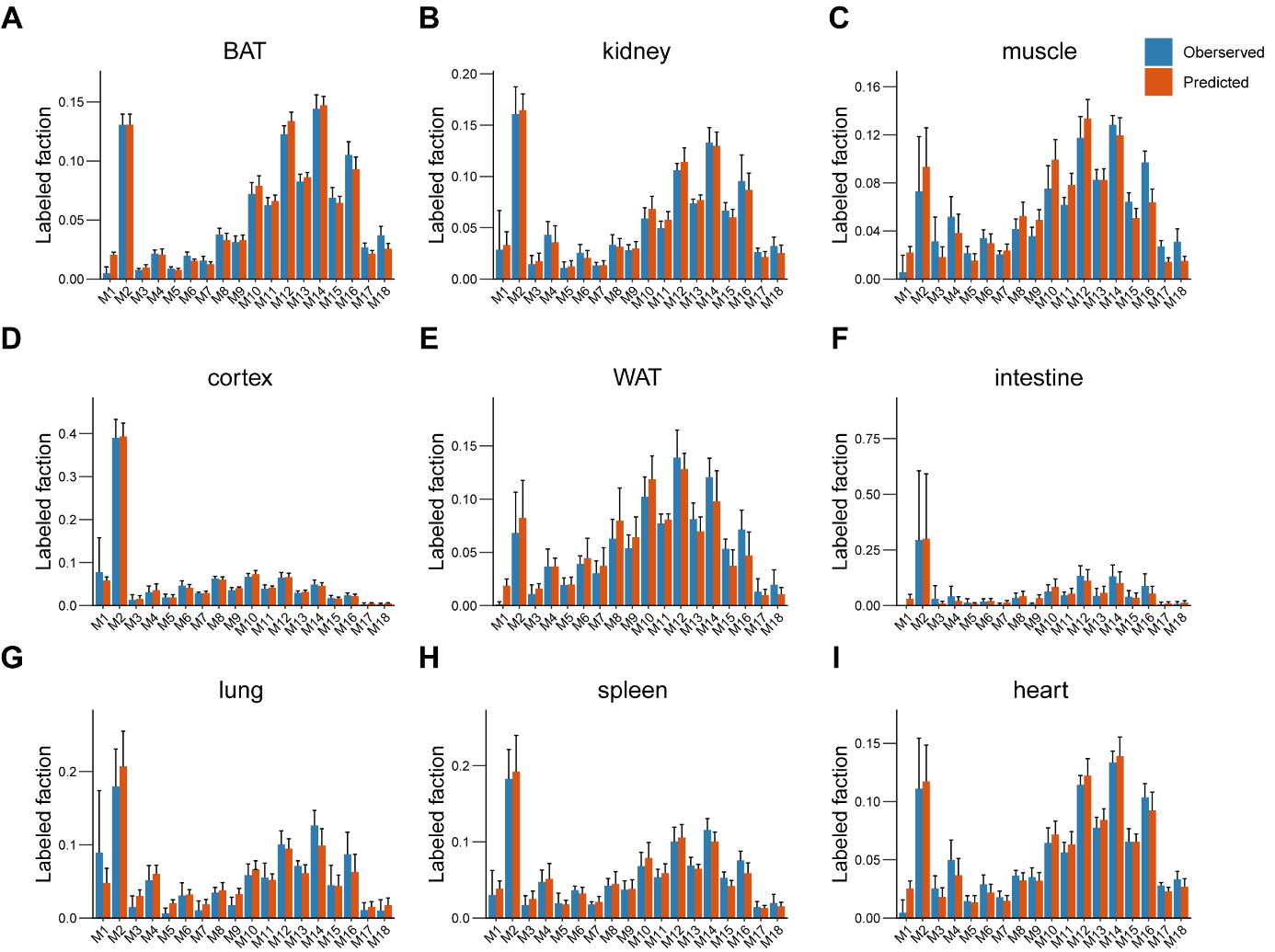


**Figure S8.** The observed MIDs and predicted MIDs of FFA (18:0) obtained from deconvolution algorithm. **A**-**I**) Comparison of observed MIDs and predicted MIDs of FFA (18:0) obtained from deconvolution algorithm in BAT, kidney, muscle, cortex, WAT, intestine, heart, lung, spleen. 24 h labeling data were used to input the model. Error bars represent the standard deviation (SD) of mean (n=6 mice; 12-week-old; C57BL/6J; male).


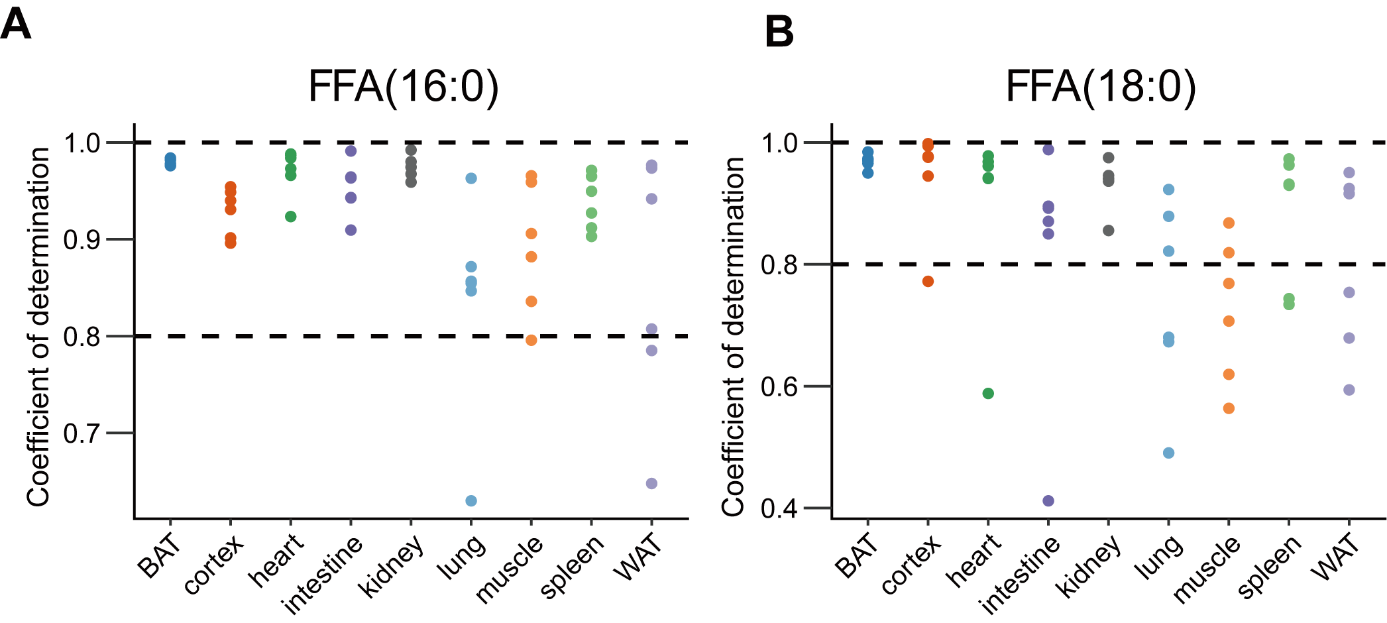


**Figure S9**. Coefficients for the MID deconvolution. **A**,**B**) Coefficients for the MID deconvolutions of FFA (16:0) and FFA (18:0) in tissues after 24 h labeling. A coefficient greater than 0.8 is considered to indicate a good fit. Each point represents a biological sample (n=6 mice; 12-week-old; C57BL/6J; male).


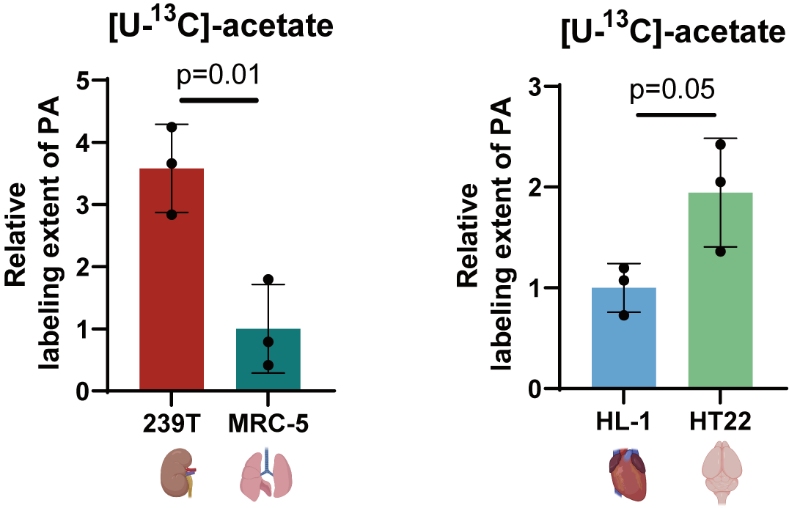


**Figure S10**. Labeling extents of FFA (16:0) in 293T, MRC-5, HL-1 and HT22 cell lines using [U-^13^C]-acetate labeling for 48 h (n=3 biologically independent replicates). The p-values were calculated by two-sided Student’s t-test.


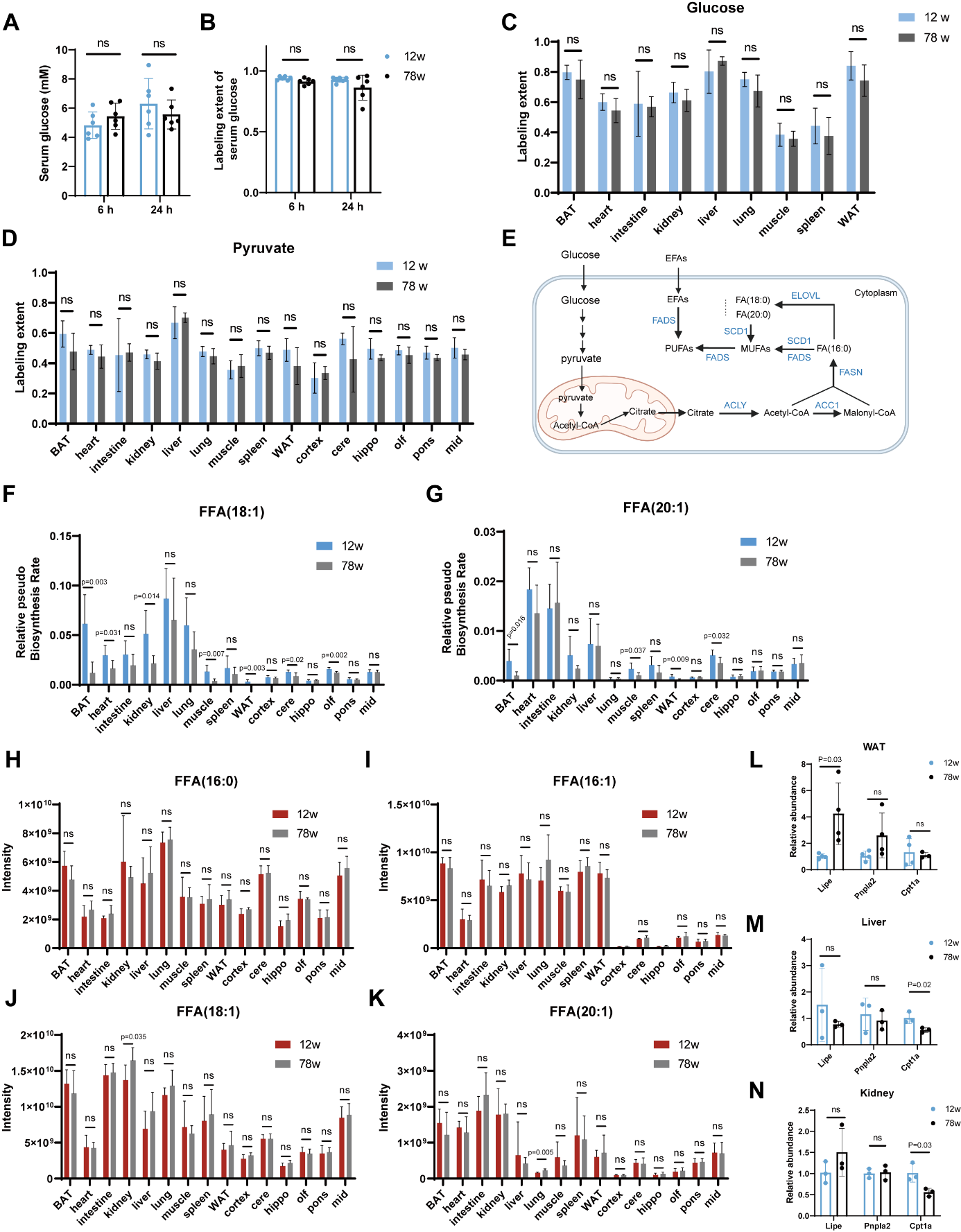


**Figure S11**. Metabolic alternations in FFAs across tissues during aging. **A**) Glucose concentrations (mM) in 12-week-old and 78-week-old mice serum after 6 h and 24 h labeling. **B**) Labeling extents of glucose in serum measured by LC-MS at 6 h and 24 h after the liquid diet refeeding. **C**,**D**) Labeling extents of glucose (C) and pyruvate (D) in young and aged mice tissues after 24 h labeling. **E**) A diagram for the fatty acid de novo synthesis pathway. EFA, essential fatty acid; MUFA, monounsaturated fatty acid; PUFA, polyunsaturated fatty acid. **F**,**G**) Bar plots show the pseudo relative biosynthesis rates of FFA (18:1) and FFA (20:1) after 24 h labeling. **H**-**K**) Bar plots show intensities of FFA (16:0), FFA (16:1), FFA (18:1), FFA (20:1) in young and aged mice tissues after 24 h labeling. All error bars represent the standard deviation (SD) of mean (n=6 for each group; C57BL/6J; male). **L**-**N**) Real-time PCR analyses of *Lipe*, *Pnpla2*, *Cpt1a* in WAT, liver, kidney. Error bars represent the standard deviation (SD) of mean (n=3-4 mice for each group;12-week-old and 78-week-old; C57BL/6J; male). All p-values were calculated by two-sided Student’s t-test.


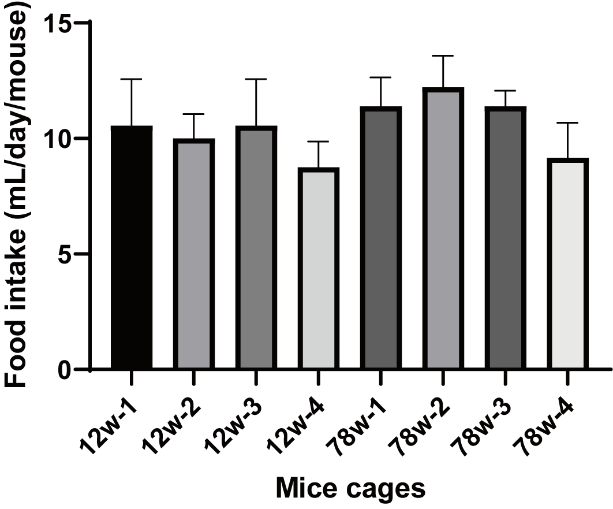


**Figure S12**. Food intake of mice during the 6-day experiment. Food intake of mice during the 6-day experiment. 3-4 mice (C57BL/6J, male) in each cage. 12w, 12-week-mice; 78w, 78-week-mice.


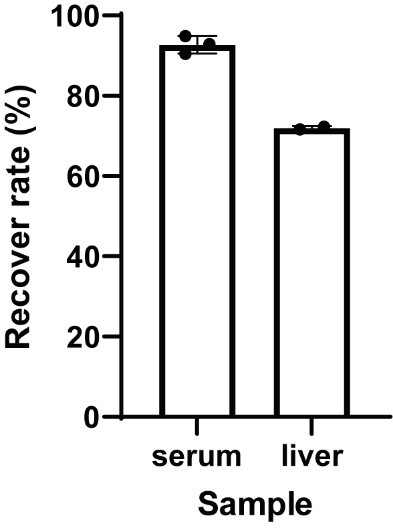


**Figure S13.** Recovery rates for FFA extraction in serum and liver samples. Recovery rates for FFA extraction in serum and liver samples were determined by spiking [U-^13^C]-FFA (18:1) into serum and liver tissue samples before and after extraction, and then analyzing the samples by LC-MS.
